# Genomic and microecological insights into the biocontrol mechanisms of *Bacillus velezensis* BER1 against rice sheath blight

**DOI:** 10.3389/fmicb.2026.1836902

**Published:** 2026-05-14

**Authors:** Zitong Kang, Sasa Zhang, Ziwei Jiao, Xiaoxing Wang, Yuquan Wei, Ruihong Wang, Yuan Chang, Ning Wang

**Affiliations:** 1 Xinjiang Key Laboratory of Lavender Conservation and Utilization, College of Biological Science and Technology, Yili Normal University, Yining, China; 2 College of Resources and Environmental Sciences, China Agricultural University, Beijing, China; 3 Organic Recycling Institute (Suzhou) of China Agricultural University, Suzhou, China; 4 Key Laboratory of Forest Ecology of Xizang Plateau, Institute of Xizang Plateau Ecology, Xizang Agriculture and Animal Husbandry University, Linzhi, China

**Keywords:** *Bacillus velezensis*, phyllosphere microbiome, *Rhizoctonia solani*, rice, whole-genome sequencing

## Abstract

Rice sheath blight is a major fungal disease threatening global rice production, causing substantial yield losses and lacking effective resistant cultivars. In this study, we systematically evaluated the biocontrol efficacy of *Bacillus velezensis* BER1 against rice sheath blight and elucidated its underlying mechanisms from three complementary perspectives: strain genomics, plant immune responses, and the phyllosphere microbiome. Genome analysis revealed that BER1 harbors 22 secondary metabolite biosynthetic gene clusters, including those encoding natural products such as fengycin and bacillaene with broad-spectrum antifungal and antibacterial activities. Application of BER1 reduced disease severity to 35.4%, outperforming conventional chemical treatment, and significantly induced the expression of rice immune-related genes (e.g., *NH1* and *PR1a*), accompanied by increased activities of defense enzymes such as POD and PAL. 16S rRNA amplicon sequencing further showed that BER1 markedly increased the relative abundance of *Bacillus* in the phyllosphere while suppressing potential pathogenic taxa, and enriched functional pathways associated with secondary metabolite biosynthesis, particularly polyketide-related pathways. Compared with chemical intervention, BER1 maintained microbial community stability through ecological niche competition and sustained metabolic activity, thereby reducing pathogen risk. Collectively, these findings provide quantitative evidence supporting BER1 as a promising green and sustainable biocontrol agent against rice sheath blight and highlight the potential of microecology-based strategies for plant disease management.

## 1 Introduction

Rice is one of the world’s major staple crops, and its yield and quality are directly linked to food security and social stability. Rice sheath blight, caused by the soil-borne fungus *Rhizoctonia solani*, is one of the most serious diseases in global rice production (Li et al., 2021; Senapati et al., 2022). It can affect plants at various growth stages, primarily infecting the leaf sheaths and leaves, and in severe cases, spreading to the panicle, leading to plant lodging and poor grain filling. Yield losses during epidemics typically range from 10 to 30%, with losses exceeding 40% in severe years (Chen et al., 2023). In the absence of efficient genetic resistance, conventional chemical control remains the primary management strategy, relying mainly on fungicides such as validamycin and carbendazim. However, the excessive reliance on chemical pesticides not only increases production costs but also poses potential risks to environmental ecosystems and human health (Pathak et al., 2022). Therefore, developing safe, environmentally friendly, and effective alternative control strategies is crucial. Biological control based on beneficial microorganisms is increasingly seen as a promising approach for the green management of rice sheath blight. Biological control enhances plant defense through direct inhibition of pathogens by antagonistic microbes or indirect mechanisms such as the induction of host plant immunity, providing a sustainable alternative to traditional chemical control (Wang et al., 2023; Tao et al., 2024).

Biological control, as a green and sustainable disease management strategy, has gained widespread attention in recent years by utilizing beneficial microorganisms and their metabolites to inhibit pathogens, induce plant immunity, or enhance plant stress resistance (Yu et al., 2022; Nguyen et al., 2025). *Trichoderma* species are among the most extensively studied fungal biocontrol agents, with their mechanisms of action including mycoparasitism, antibiotic production, nutrient/space competition, and the induction of systemic resistance (ISR). *Trichoderma* directly infects and degrades the hyphae of pathogens, secreting various antifungal metabolites (such as volatile organic compounds and alkaloids) to inhibit pathogen growth, while also interacting with the host plant to activate signaling pathways such as jasmonic acid and ethylene, thereby enhancing the plant’s defense response and promoting nutrient uptake (Mukherjee et al., 2022; Yao et al., 2023). This leads to broad-spectrum control of various crop diseases. *Pseudomonas* species control pathogens through the production of various antimicrobial compounds (such as phenazine antibiotics, cyanide, and 2,4-diacetylphenylalanine), while also competing for resources like iron and inducing host immunity to enhance defense. These mechanisms have been experimentally validated in the control of various soil-borne and sclerotial diseases (Walsh et al., 2001; Dobrzyński and Jakubowska, 2025). *Streptomyces* species from the phylum Actinobacteria are also important biocontrol agents, primarily producing large quantities of antifungal and antibacterial antibiotics, chitinases, and glycoside hydrolases, which break down pathogen cell structures and inhibit their metabolic activity, while also promoting plant health and nutrient cycling (Nazari et al., 2023; Ali et al., 2024). *Bacillus subtilis* and *Bacillus velezensis* are widely studied due to their spore-forming stability, strong environmental adaptability, and ease of industrial production. Their direct actions mainly include antimicrobial competition, antibiotic and metabolite secretion, and physical suppression of pathogens. *Bacillus* species produce a range of secondary metabolites (such as lipopeptides, bacillomycin, fengycin, and surfactin), which exert bactericidal or bacteriostatic effects by disrupting pathogen cell membranes and inhibiting hyphal growth, demonstrating significant inhibitory effects against various fungal pathogens (Zihalirwa Kulimushi et al., 2017; Sarwar et al., 2018). The disease resistance characteristics of *B. subtilis* lie in its ability to produce antifungal metabolites and antimicrobial proteins that directly block pathogen growth, while further inhibiting pathogen colonization and proliferation by competing for space and nutrients in the rhizosphere (Zhang et al., 2023). *B. velezensis* can synthesize various bioactive compounds, including polyketides and lipopeptides, which enable it to display broad-spectrum antifungal activity against fungi such as *Fusarium* spp. and *Rhizoctonia* spp. (Fazle Rabbee and Baek, 2020). In addition to direct mechanisms, these biocontrol strains can activate plant defense responses through indirect mechanisms, enhancing host resistance. Studies have shown that *B. velezensis* not only exerts direct antagonism by producing antimicrobial substances but also induces systemic resistance (ISR), activating host immune pathways, increasing the activity of disease-related enzymes (such as peroxidases and phenylalanine ammonia-lyase), and enhancing the host’s response to hormonal signals (such as salicylic acid and jasmonic acid), thereby boosting the plant’s overall defense capacity (Yu et al., 2022; Chen et al., 2024). Furthermore, bacterial volatile organic compounds (such as 2,3-butanediol) can act as signaling molecules to induce stomatal closure and chemical defense responses, indirectly blocking pathogen invasion and promoting disease resistance (Wu et al., 2018; Montejano-Ramírez et al., 2024). Despite numerous reports on the screening and efficacy evaluation of biocontrol agents, the systematic functional characterization of these beneficial strains, their disease control mechanisms, and the intrinsic links with host plant physiological responses remain insufficiently explored. Current research primarily focuses on describing antagonistic activity, lacking comprehensive insights into disease-related functional genes from a whole-genome perspective, and the integration of plant defense responses, microbial community dynamics, and multi-scale data to fully reveal their biocontrol mechanisms. Additionally, the dynamic changes in plant phyllosphere microbiomes, as a critical interface between plants and the environment, play a key role in disease control and plant health, yet have been rarely considered in previous biological control studies.

Based on the above background, this study aims to screen and identify biocontrol strains with excellent disease resistance potential, and systematically analyze their functional gene composition and disease-related genetic basis using whole-genome sequencing technology. A pot experiment with rice will be conducted to evaluate the efficacy of these biocontrol strains in controlling rice sheath blight, assessing their impact on the disease-related enzyme activity and gene expression in rice leaves. Additionally, the study explored the changes in the phyllosphere microbiome structure and functional predictions following the application of biocontrol strains. This research will provide foundational support for optimizing biocontrol applications and developing green management strategies for rice sheath blight.

## 2 Materials and methods

### 2.1 Comparison of enzyme production, indole acetic acid production, and antagonistic activity against pathogens of biocontrol strains

The biocontrol strains tested in this study were sourced from the Institute of Organic Recycling at China Agricultural University (Suzhou). The strains were initially retrieved from a −80°C freezer, activated on LB agar plates at 28°C, and then inoculated into 5 mL of LB liquid medium, followed by incubation at 30°C with shaking at 180 rpm for 24 h. The strains were obtained from a laboratory collection and were not taxonomically identified; species-level identification was performed only after the screening process, and only for strains exhibiting significant functional activity. The bacterial suspension was adjusted to an OD_600_ of 0.05. Enzyme production ability was assessed by inoculating 5 μL of the bacterial suspension onto agar plates containing media for protease, chitinase, glucanase, and siderophore-producing enzyme activity (Roberts and Selitrennikoff, 1988; Shin et al., 2001; Kurniawati et al., 2020; Pandey, 2023). The plates were incubated at 28°C for 48 h, and the ratio of the radius of the clear zone (R) to the radius of the bacterial growth (r) was measured. Indole acetic acid (IAA) production was determined using the method of Sarwar and Kremer (1995), with the IAA concentration calculated based on the OD_530_ values and a standard curve. Antagonistic activity against fungal pathogens was evaluated by inoculating the pathogens onto PDA plates, transferring 6-mm mycelial plugs to fresh media, and applying 5 μL of the biocontrol strain suspension using a micropipette at four equidistant points around the fungal plug, at an approximate distance of 2.5 cm from the pathogen. After 4 days of incubation, the size of the antagonistic zone was measured, and the antagonistic effect was calculated using the (R-r)/R ratio (r represents the radius of the fungal colony, and R represents the distance from the bacterial colony edge to the center of the fungal colony).

### 2.2 Whole-genome sequencing and analysis of the strain

After activation on LB agar plates, the strains were inoculated into 50 mL of LB liquid medium and incubated at 28°C with shaking at 130 rpm for 24 h. Genomic DNA was then extracted and quantified. Whole-genome sequencing was performed using both the PacBio Sequel IIe and Illumina platforms. The Illumina library was constructed with DNA fragments of 400 bp, and paired-end sequencing (2 × 150 bp) was carried out. Quality control was performed using fastp v0.23.0 to remove low-quality reads. The data were assembled using Unicycler v0.4.8, and genome correction was performed with Pilon v1.22. Gene prediction was conducted using Glimmer, GeneMarkS, and tRNAscan-SE. Functional annotation was carried out using BLASTP, Diamond, HMMER, and other tools, with annotations based on the COG, KEGG, and GO databases. Finally, secondary metabolite gene clusters were predicted using antiSMASH v5.1.2.

### 2.3 Design of pot experiment

To evaluate the efficacy of *Bacillus velezensis* BER1 in controlling rice sheath blight, three treatment groups were established: control with water (CK), validamycin (J), and *Bacillus velezensis* BER1 (B) (1 × 10^8^ CFU/mL), with three replicates for each treatment. Validamycin and the bacterial suspension were applied at a 1,000-fold dilution. Treatments were applied twice, 10 days apart over a 20-day period. During inoculation, the treatment was applied until it dripped from the rice plants. Prior to spraying, the pots were filled with water to prevent root absorption. On day 21, rice plants were inoculated by spraying a spore suspension of *Rhizoctonia solani* (1 × 10^5^ spores mL^−1^) onto the aerial parts of the plants until runoff. Prior to inoculation, pots were filled with water, which was drained immediately after spraying to minimize uptake through the roots. After inoculation, plants were placed in a growth chamber at 30°C with a 16 h light/8 h dark photoperiod and relative humidity > 90% for 72 h to facilitate infection. Subsequently, plants were returned to normal conditions (28°C, 16 h light/8 h dark, relative humidity 60%).

### 2.4 Measurement of defense enzyme activity and disease resistance gene expression in rice leaves

Rice leaves were collected at 24, 48, and 72 h after pathogen inoculation to measure the contents of peroxidase (POD), phenylalanine ammonia-lyase (PAL), and catalase (CAT). The assays were conducted using Nanjing Jiancheng Bioengineering kits according to the manufacturer’s instructions. Concurrently, leaf samples were collected for analysis of disease resistance gene expression. Total RNA was extracted using a commercial RNA extraction kit and treated with DNase I to remove genomic DNA. RNA quality was assessed using a NanoDrop spectrophotometer, and integrity was evaluated by agarose gel electrophoresis. The A_260_/_280_ ratio was between 1.8 and 2.1, with clear bands observed. cDNA was synthesized using a TaKaRa kit and amplified in a CFX96 real-time fluorescence PCR system. The reaction mixture (25 μL) contained SYBR Green Master Mix, primers, and cDNA template. The real-time PCR program consisted of an initial denaturation at 95°C for 3 min, followed by 40 cycles of 95°C for 10 s and 60°C for 30 s, with a melting curve analysis conducted at the end. Gene expression levels were normalized using the *Actin* gene as an internal reference. Primer sequences are provided in Supplementary Table 1 (Liu et al., 2005; Wang et al., 2006; Zhao et al., 2008).

### 2.5 Phyllosphere microbiome analysis

Twenty days after treatment, rice leaves were collected using scissors to cut leaves that had not contacted the ground or hands, and placed into 50 mL centrifuge tubes. The leaf samples were then randomly mixed. For microbial enrichment, physiological saline was added, and the samples were shaken at 50–60 Hz for 30 s. After centrifugation at 10,000 rpm for 10 min at 4°C, the supernatant was discarded. The pellet was snap-frozen in liquid nitrogen and stored at −80°C. The samples were shipped on dry ice to Shenzhen Megabio Biotechnology Co., Ltd. for 16S rRNA amplicon sequencing analysis. After genomic DNA extraction, the integrity, concentration, and purity of the DNA were assessed by agarose gel electrophoresis and NanoDropOne. PCR amplification was performed using barcode-labeled primers 338F (ACTCCTACGGGAGGCAGCA) and 806R (GGACTACHVGGGTWTCTAAT) with PremixTaq (TaKaRa). After gel recovery, the library was constructed and sequenced using the HiSeq or MiSeq platform. The sequencing results were stored in FASTQ format.

### 2.6 Pot experiment evaluation of efficacy against rice sheath blight

The disease severity was recorded on days 7, 14, 21, and 28 after inoculation with the pathogen. Each treatment had three biological replicates. Disease severity was classified according to the GB/T 15791-2011 standard, with specific criteria provided in Supplementary Table 2. The disease severity was calculated using the formula: Disease Severity = (Disease Grade × Number of Plants at That Grade)/(Total Number of Plants × Highest Disease Grade) × 100%. The efficacy of the treatment was calculated using the formula: Efficacy = [(Disease Severity of Control Group − Disease Severity of Treatment Group)/Disease Severity of Control Group] × 100%.

### 2.7 Data analysis

All experimental data were recorded and organized using Microsoft Excel 2021, and graphs were generated using Origin 2023b. Differences between treatments were evaluated using the Least Significant Difference (LSD) test (*p* < 0.05). Alpha diversity indices, including Chao1 richness and Shannon diversity, were calculated, and beta diversity was assessed based on Bray–Curtis dissimilarity, visualized by Principal Coordinate Analysis (PCoA). The community structure differences were tested using Permutational Multivariate Analysis of Variance (PERMANOVA). Gene expression levels were calculated using the 2∧−ΔΔCt method. Microbial community functional predictions and differential analysis were performed using the PICRUSt2 tool. Raw data were processed with Cutadapt to remove adapter sequences and quality-controlled in QIIME2. Feature sequences (ASVs) were constructed using the DADA2 plugin. Community diversity was calculated using QIIME2 and R, with PERMANOVA used for significance testing. ASV sequences were compared to a reference phylogenetic tree, and gene family abundance (KO level) was inferred using the Hidden-state prediction algorithm. KO abundance was summarized to the KEGG Pathway level for metabolic pathway analysis. KO and KEGG pathway abundance data were normalized within samples, and gene set enrichment analysis was performed using the fgsea algorithm. Microbial community phenotypic predictions were performed using the BugBase platform, and differential microbial populations were identified using linear discriminant analysis effect size (LEfSe) and differential expression analysis based on the negative binomial distribution implemented in DESeq2. All statistical analyses were conducted in the R environment, and graphs were generated using R packages including ggplot2, ggpicrust2, and dplyr.

## 3 Results

### 3.1 Comparison of enzyme production, IAA production, and antagonistic activity against pathogens of biocontrol strains

To evaluate the disease resistance potential of different biocontrol strains, this experiment compared the performance of 10 strains in terms of enzyme activity (The radii of the clear zone (R) and bacterial colony (r) were measured. The R/r ratio was used to evaluate relative enzymatic activity, with higher values indicating stronger enzyme production and substrate degradation), IAA production, and pathogen inhibition (r represents the radius of the fungal colony, and R represents the distance from the bacterial colony edge to the fungal center. The (R−r)/R ratio indicates inhibitory activity, with higher values reflecting stronger antagonism). In this experiment, the strains XY21 (2.15), 92 (2.20), and BER1 (2.08) exhibited the highest protease activity. For siderophore production, strains 233 (2.30), 92 (2.33), and HH4 (2.00) demonstrated strong capabilities. In terms of glucanase production, BER1 showed the highest activity at 3.36, followed by KC21 (2.49) and 424 (2.41). For chitinase production, XY21 exhibited the highest activity at 1.58, followed by 7ZE3 (1.49) and BER1 (1.49). In IAA production, LUU (18.46 mg/L) exhibited the highest level, followed by XY21 (16.95 mg/L), H83 (15.82 mg/L), and BER1 (14.56 mg/L). The inhibition of pathogens varied among the strains, with BER1 showing the most significant inhibitory effects on *Fusarium oxysporum* f. sp. *cubense*, *Rhizoctonia solani*, *Botrytis cinerea*, *Fusarium oxysporum* f. sp. *niveum*, and *Phytophthora infestans*, making BER1 the strain selected for subsequent experiments (Table 1).

**TABLE 1:** Enzyme production, IAA synthesis, and antagonistic activity of bacterial strains against plant pathogens.

Strain	Proteinase	Siderophore	Glucanase	Chitinase	IAA mg/L	*F. oxysporum* f. sp. *cubense*	*R. solani*	*B. cinerea*	*F. oxysporum* f. sp. *niveum*	*P. infestans*
KC21	1.92 ± 0.13 bcd	1.57 ± 0.07 c	2.49 ± 0.11 b	1.25 ± 0.09 d	13.26 ± 0.36 e	0.30 ± 0.05 b	0.13 ± 0.02 d	0.24 ± 0.03 c	×	0.39 ± 0.05 a
XY21	2.15 ± 0.11 ab	1.62 ± 0.08 c	1.76 ± 0.11 d	1.58 ± 0.08 a	16.95 ± 0.45 b	0.17 ± 0.04 c	×	0.34 ± 0.09 b	0.22 ± 0.03 c	×
92	2.20 ± 0.18 a	2.33 ± 0.13 a	×	×	8.38 ± 0.28 gh	×	0.13 ± 0.01 d	0.33 ± 0.04 b	×	0.33 ± 0.07 a
233	1.45 ± 0.18 e	2.30 ± 0.12 a	×	1.33 ± 0.10 cd	4.52 ± 0.32 i	0.16 ± 0.03 c	0.26 ± 0.04 c	×	0.20 ± 0.03 c	×
7ZE3	1.86 ± 0.11 cd	1.45 ± 0.10 c	1.89 ± 0.09 cd	1.49 ± 0.10 ab	10.42 ± 0.32 f	×	×	0.33 ± 0.05 b	0.27 ± 0.04 c	×
HH4	2.12 ± 0.17 ab	2.01 ± 0.11 b	×	1.42 ± 0.06 bc	7.91 ± 0.31 h	0.17 ± 0.04 c	0.35 ± 0.06 b	×	×	0.16 ± 0.08 b
424	1.86 ± 0.16 cd	×	2.41 ± 0.07 b	×	8.79 ± 0.29 g	0.31 ± 0.05 b	0.15 ± 0.03 d	×	0.36 ± 0.05 b	0.19 ± 0.04 b
H83	1.09 ± 0.19 f	×	×	×	15.82 ± 0.42 c	×	×	0.17 ± 0.01 d	0.21 ± 0.03 c	0.23 ± 0.03 b
LUU	1.68 ± 0.14 de	×	2.09 ± 0.08 c	×	18.46 ± 0.46 a	×	0.33 ± 0.04 b	0.16 ± 0.03 d	×	0.19 ± 0.04 b
BER1	2.08 ± 0.12 abc	1.51 ± 0.11 c	3.36 ± 0.09 a	1.49 ± 0.06 ab	14.56 ± 0.36 d	0.59 ± 0.08 a	0.57 ± 0.05 a	0.61 ± 0.02 a	0.69 ± 0.08 a	0.35 ± 0.05 a

Values are presented as mean ± standard deviation (SD) (*n* = 3). Different letters within the same column indicate significant differences among strains (*p* < 0.05). CK, control; J, pesticide treatment; B, biocontrol treatment; IAA, indole-3-acetic acid. The symbol “×” indicates no detectable activity.

### 3.2 Genomic analysis of *Bacillus velezensis* BER1

**FIGURE 1:**
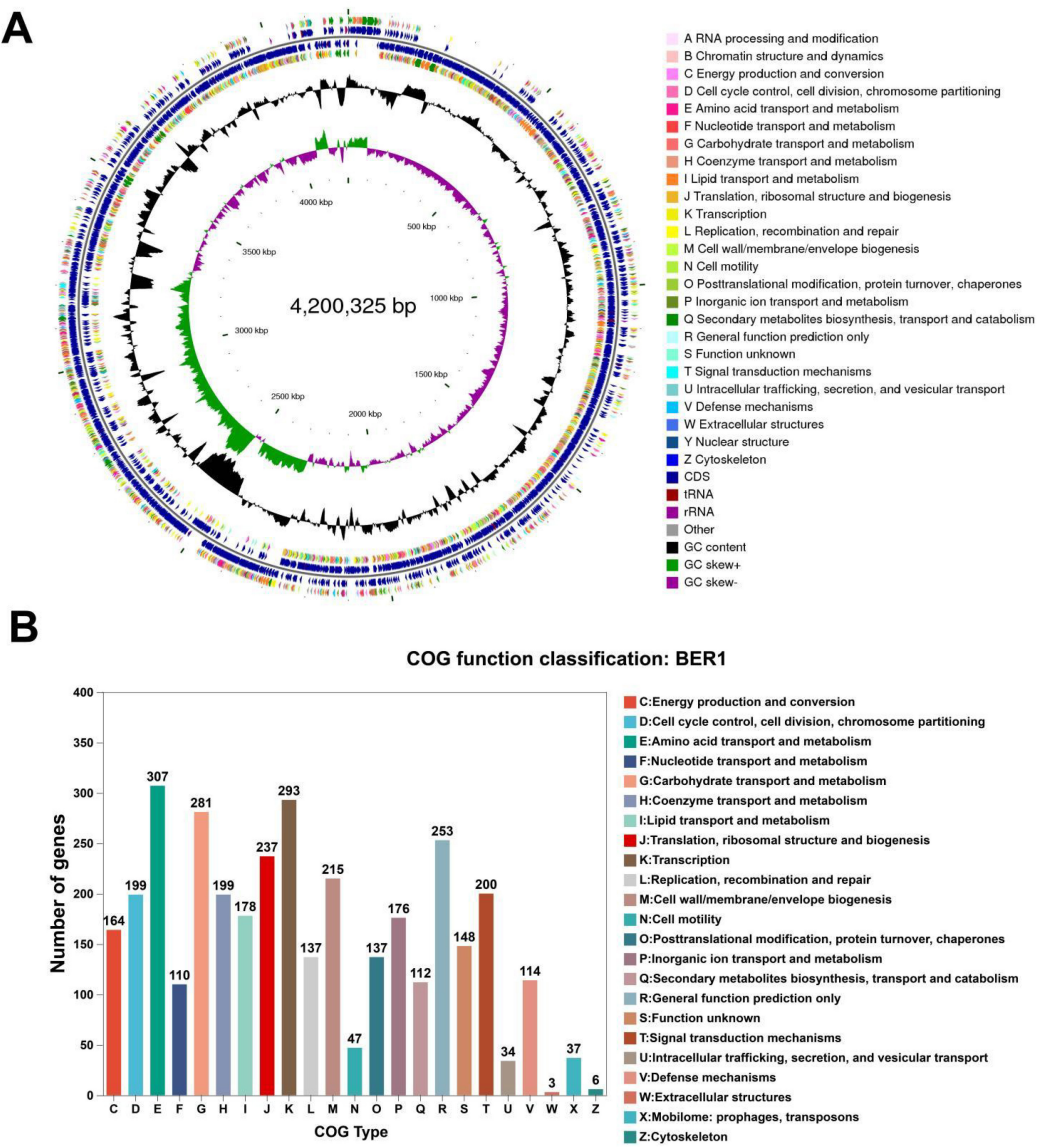
**(A)** Genomic characteristics and circular genome map of *Bacillus velezensis* BER1. The circles, from outer to inner, represent the forward strand coding sequences, tRNA and rRNA, reverse strand coding sequences, tRNA and rRNA, GC content, and GC skew. **(B)** Classification of the number of homologous gene clusters in the genome.

To further analyze the genomic characteristics and functions of the strain BER1, a detailed annotation and functional analysis of its genome were conducted in this study. The complete genome of *Bacillus velezensis* BER1 consists of a 4,200,325 bp chromosome, which is made up of 27 scaffolds, with a Scaffold N50 of 573,477 bp and an average GC content of 45.83%. The genome contains 4,156 coding sequences (CDS), including 85 tRNA genes and 9 rRNA genes. The intergenic region occupies 10.54% of the genome, with a GC content of 38.43%. The average gene length is 904.16 bp, and the average GC content of the genes is 46.71%. A total of 105 tandem repeat sequences (TRs) were identified (Figure 1A). Homologous genes within the genome were annotated and classified by comparison with the EggNOG database. The results showed that 3,198 genes were annotated and classified into 23 categories. As illustrated in Figure 1B, the annotated genes include 307 involved in amino acid transport and metabolism, 110 in nucleotide transport and metabolism, 281 in carbohydrate transport and metabolism, 199 in coenzyme transport and metabolism, 178 in lipid transport and metabolism, 237 in translation/ribosomal structure and biosynthesis, 293 in transcription, 215 in cell wall/membrane/envelope biogenesis, and 200 in signal transduction mechanisms.

**FIGURE 2:**
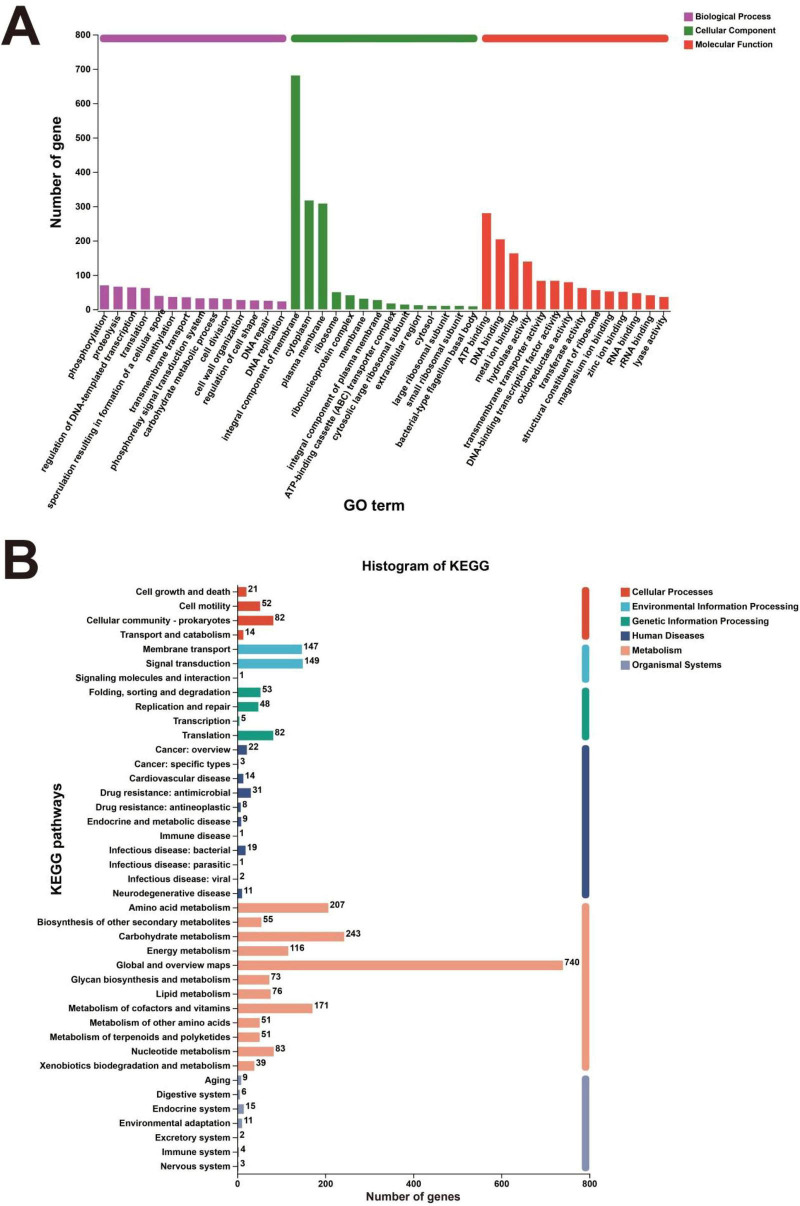
GO annotation of *Bacillus velezensis* BER1 genes in the GO database **(A)**; pathway enrichment in the KEGG database **(B)**.

By comparing the protein sequences encoded by BER1 with the GO database, GO annotations were obtained, and functional clustering analysis was performed. The analysis was categorized into three main aspects: biological processes, cellular components, and molecular functions. The strain BER1 was found to have 1,218 genes associated with cellular components, 1,960 genes associated with molecular functions, and 1,281 genes associated with biological processes (Figure 2A). The KEGG database annotated a total of 2,730 genes (Figure 2B), of which 1,905 genes, accounting for 69.78%, were related to metabolic processes. Specifically, within metabolic functions, the largest number of genes was found in the “Global and overview maps” category (740 genes), followed by carbohydrate metabolism (243 genes), amino acid metabolism (207 genes), cofactor and vitamin metabolism (171 genes), energy metabolism (116 genes), nucleotide metabolism (83 genes), lipid metabolism (76 genes), and sugar biosynthesis and metabolism (73 genes) (Figure 2B).

After conducting antiSMASH analysis on the whole genome of *Bacillus velezensis* BER1, a total of 22 secondary metabolite biosynthetic gene clusters (BGCs) were identified, involving multiple types of natural product synthesis pathways (Table 2). These BGCs include categories such as transAT-PKS-like, T3PKS, terpene, NRPS, lanthipeptide, bacteriocin, PKS-like, and NRPS-like. Among them, clusters 4, 5, 6, 8, 10, 11, and 12 showed high similarity to known natural product gene clusters, with predicted products including fengycin (100% similarity), bacillaene (100%), macrolactin H (100%), bacilysin (100%), subtilin (100%), bacillibactin (100%), and surfactin (82%). Additionally, products such as difficidin, rhizocticin A, and butirosin A/B were identified in some clusters, although their similarity was lower or they were unannotated. Specifically, several BGCs were predicted to belong to polyketide synthesis (transAT-PKS/T3PKS) and nonribosomal peptide synthesis (NRPS) pathways, indicating that BER1 possesses the genetic basis for synthesizing a variety of antimicrobial secondary metabolites. Furthermore, gene clusters related to terpenes, siderophores, and glycopeptide antibiotics were also identified.

**TABLE 2:** Predicted secondary metabolite biosynthetic gene clusters of *Bacillus velezensis* BER1 identified by antiSMASH.

Cluster ID	Type	Length (bp)	Similar cluster	Similarity (%)	Gene no.
Cluster1	transAT-PKS-like	46,370	Difficidin	53	30
Cluster2	T3PKS	39,996	−	−	62
Cluster3	Terpene	21,885	−	−	22
Cluster4	NRPS	125,767	Fengycin	100	62
Cluster5	transAT-PKS-like	108,892	Bacillaene	100	52
Cluster6	transAT-PKS	88,222	Macrolactin H	100	44
Cluster7	transAT-PKS	34,151	Difficidin	46	28
Cluster8	Other	41,420	Bacilysin	100	43
Cluster9	NRPS	23,291	−	−	21
Cluster10	Lanthipeptide	26,811	Subtilin	100	29
Cluster11	Bacteriocin	51,791	Bacillibactin	100	46
Cluster12	NRPS	65,094	Surfactin	82	40
Cluster13	transAT-PKS	46,712	Rhizocticin A	6	25
Cluster14	PKS-like	41,246	Butirosin A / butirosin B	7	41
Cluster15	Terpene	20,742	−	−	22
Cluster16	NRPS-like	22,506	Rhizocticin A	16	20
Cluster17	transAT-PKS-like	23,946	Difficidin	26	4
Cluster18	NRPS	9,094	Fengycin	13	2
Cluster19	NRPS	8,262	−	−	2
Cluster20	NRPS	6,890	−	−	1
Cluster21	NRPS	6,740	−	−	2
Cluster22	NRPS	2,183	−	−	2

“–” indicates no similar known gene cluster. Similarity (%) represents the similarity between the predicted gene cluster and known clusters in the antiSMASH database.

### 3.3 Effects of different treatments on disease control and severity in rice plants

**FIGURE 3:**
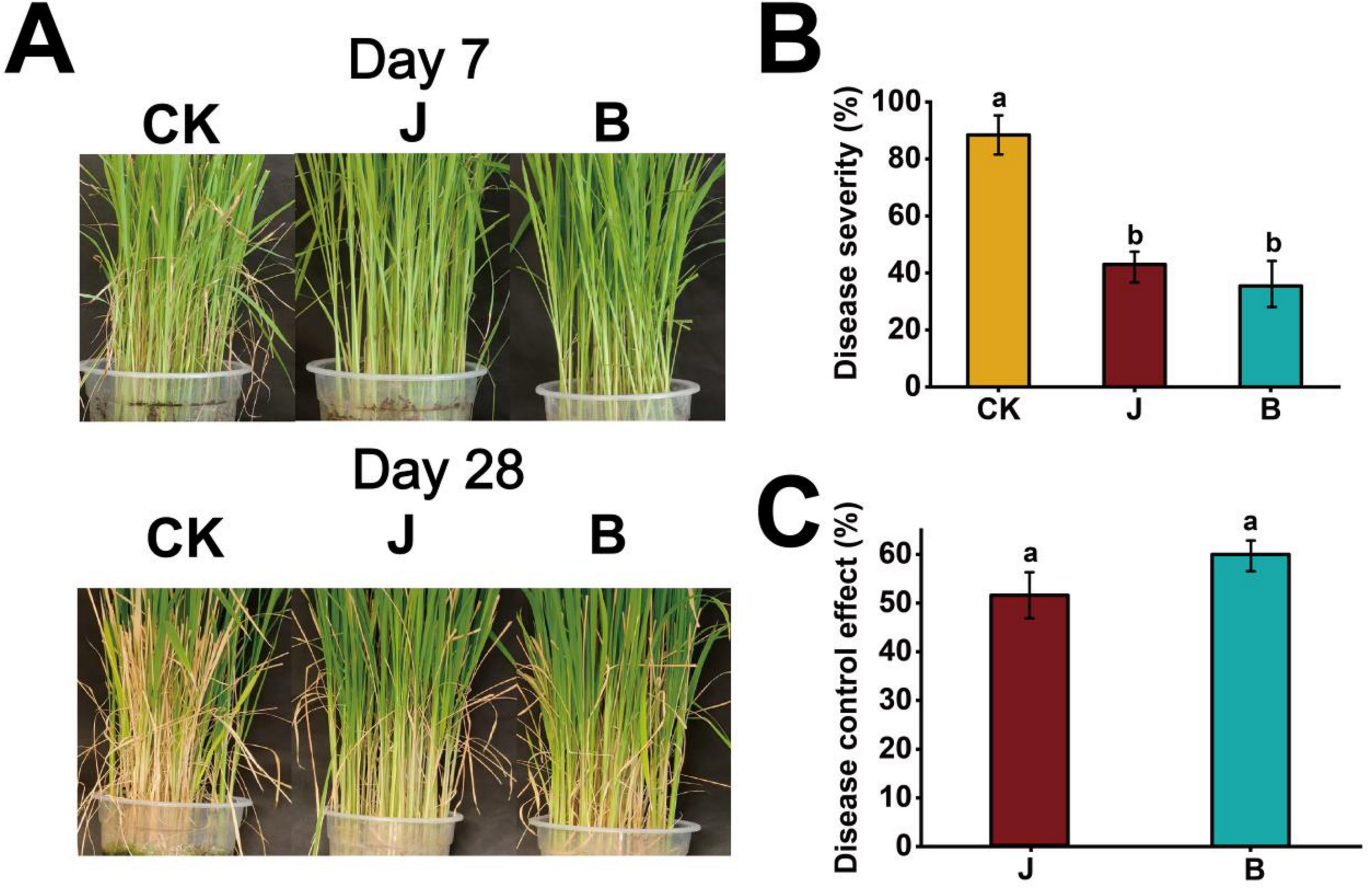
Evaluation of disease severity and control efficacy in rice under different treatments. **(A)** Phenotypic comparison of rice plants at 7 and 28 days after pathogen inoculation under different treatments: CK (control), J (chemical treatment), and B (biocontrol treatment). **(B)** Disease severity (%) under different treatments. **(C)** Disease control efficacy (%) under different treatments. Values are presented as mean ± standard deviation (SD) (*n* = 3). Different letters above bars indicate significant differences among treatments (*p* < 0.05) based on one-way ANOVA followed by the LSD test.

To evaluate the impact of different treatments on rice disease control, this experiment compared the effects of J, B, and CK treatments on disease severity. The results showed that the treatments significantly affected both disease development and control efficacy. On day 7, rice plants in the CK group exhibited typical sheath blight symptoms, including elongated, water-soaked lesions on leaf sheaths and initial chlorosis, whereas plants in the J and B treatment groups showed fewer and smaller lesions with limited disease progression. By day 28, disease symptoms in the CK group were markedly aggravated, characterized by extensive lesion expansion, leaf blight, and wilting of lower leaves. In contrast, plants treated with J and B displayed significantly reduced lesion size and number, with greener leaves and less wilting, indicating effective suppression of disease development (Figure 3A). In terms of disease severity, the CK treatment had a severity of 88.4%, while the J and B treatments had severity levels of 43.1 and 35.4%, respectively (Figure 3B). Disease control efficacy analysis showed that the control effectiveness of the J and B treatments was 60.0 and 51.2%, respectively, indicating that both treatments effectively reduced disease severity (Figure 3C). The control efficacy of the CK group was defined as 0% according to the calculation formula and was therefore not included in the figure.

### 3.4 Changes in expression of rice disease defense-related genes and enzyme activity

**FIGURE 4:**
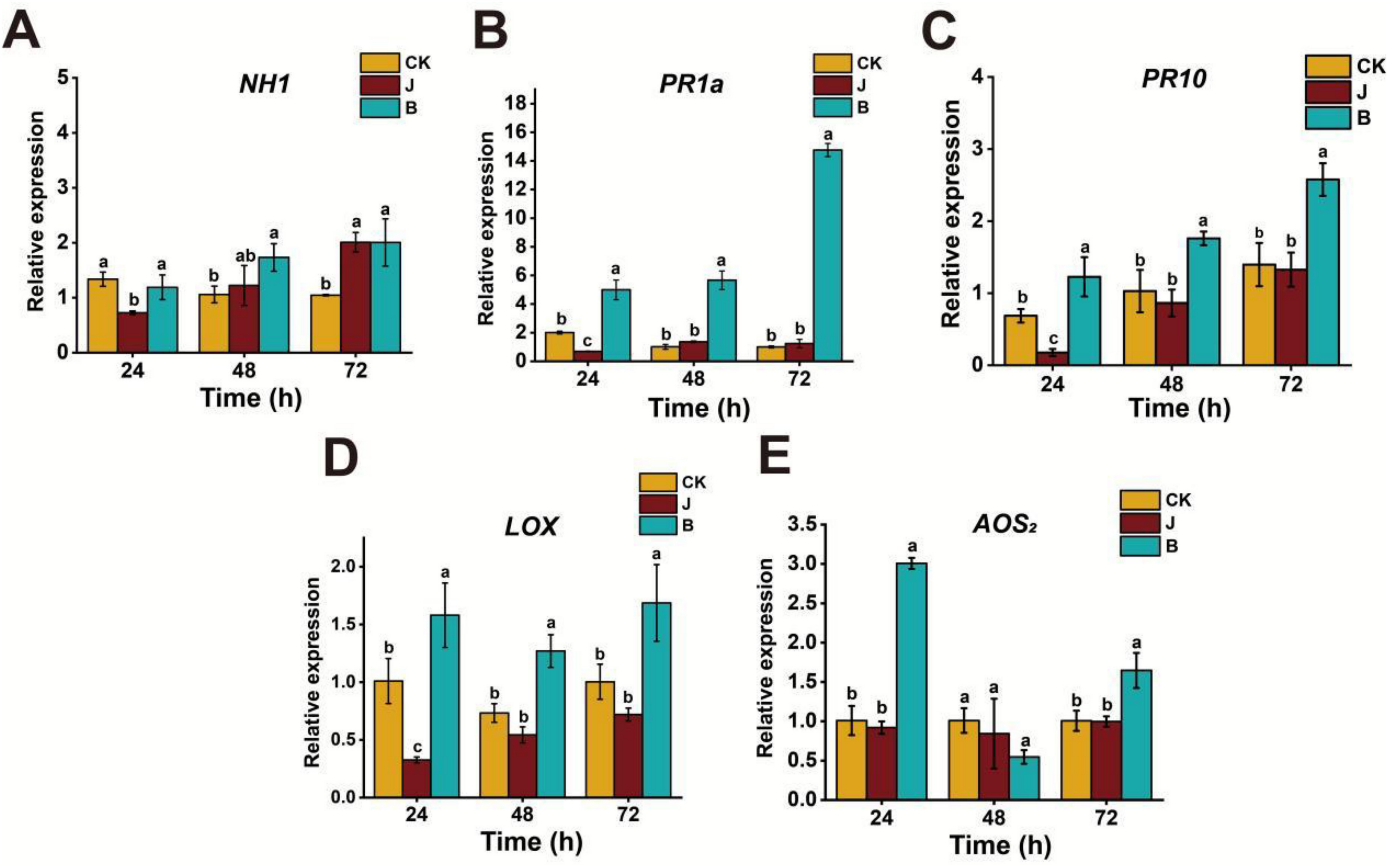
Quantification of the relative expression levels of *NH1* **(A)**, *PR1a* **(B)**, *PR10* **(C)**, *LOX* **(D)**, and *AOS*_2_ **(E)** Genes by qRT-PCR, normalized to the reference gene. The bar graphs represent the mean values, with different letters indicating significant differences based on one-way ANOVA and LSD multiple comparison test (*p* < 0.05).

To investigate the effects of different treatments on rice gene expression and enzyme activity, this experiment analyzed the changes in gene expression (The reference gene was Actin) and enzyme activity under B, J, and CK treatments. The results indicated that the treatments significantly affected the expression of relevant genes in rice. The B treatment exhibited a significant induction effect on the relative expression of multiple genes. For the *NH1* gene, the expression in the B treatment was higher than in the J treatment at 24 h, and at 48 h, it was higher than in the other treatments. At 72 h, the expression remained high but was not significantly different from the J treatment (Figure 4A). The expression of *PR1a* and *PR1*0 genes in the B treatment was significantly higher than in the CK and J treatments from 24 to 72 h, indicating a strong induction effect (Figures 4B,C). The expression of the *LOX* gene changed over time, but from 24 to 72 h, the B treatment showed significantly higher expression than the other treatments (Figure 4D). For the *AOS*_2_ gene, the expression was highest in the B treatment at 24 h. At 48 h, the expression decreased, with no significant difference from the J and CK treatments, but at 72 h, the expression in the B treatment significantly increased (Figure 4E).

The experimental results showed significant changes in the activity of POD, CAT, and PAL enzymes at different time points. POD activity in the B treatment was significantly higher than that in the CK and J treatments at 24, 48, and 72 h, with the peak activity of 27.44 U/mL observed at 48 h, which was markedly higher than the 11.78 U/mL in the CK treatment and 21.84 U/mL in the J treatment. For CAT, the activity in the J and B treatments was higher than that in the CK treatment at 24 h, with B treatment showing values of 6.38 U/mL and 7.21 U/mL at 24 and 48 h, respectively, both higher than 5.90 U/mL and 4.06 U/mL in the CK treatment. At 72 h, CAT activity was highest in the J treatment at 9.16 U mL^−1^, followed by the B treatment at 7.43 U mL^−1^, and lowest in the CK group at 4.48 U mL^−1^. As for PAL, the activity in the B treatment was significantly higher than in the other treatments from 24 to 72 h, with the highest activity of 45.36 U/mL at 72 h, significantly higher than the 20.34 U/mL in the CK treatment and 39.80 U/mL in the J treatment (Table 3).

**TABLE 3:** POD, CAT, and PAL enzyme activities in rice leaves under different treatments (24–72 h).

Time (h)	Treatment	POD (U/mL)	CAT (U/mL)	PAL (U/mL)
24	CK	9.00 ± 0.20 c	5.90 ± 0.11 c	22.59 ± 0.53 c
	J	17.07 ± 0.46 b	9.16 ± 0.15 a	24.39 ± 1.03 b
	B	20.79 ± 0.37 a	6.38 ± 0.28 b	41.28 ± 0.97 a
48	CK	11.78 ± 0.63 c	4.06 ± 0.23 c	22.55 ± 1.11 c
	J	21.84 ± 1.16 b	9.60 ± 0.20 a	34.86 ± 1.00 b
	B	27.44 ± 0.79 a	7.21 ± 0.26 b	44.81 ± 0.81 a
72	CK	11.99 ± 0.53 c	4.48 ± 0.05 c	20.34 ± 0.79 c
	J	14.46 ± 0.84 b	9.16 ± 0.11 a	39.80 ± 0.99 b
	B	22.62 ± 0.57 a	7.43 ± 0.12 b	45.36 ± 1.49 a

Values are presented as mean ± standard deviation (SD) (*n* = 3). CK, control; J, pesticide treatment; B, biocontrol treatment. Different letters within the same column indicate significant differences among treatments at the same time point (*p* < 0.05), as determined by one-way ANOVA followed by the LSD test.

### 3.5 Effects of different treatments on the phyllosphere bacterial community structure of rice

**FIGURE 5:**
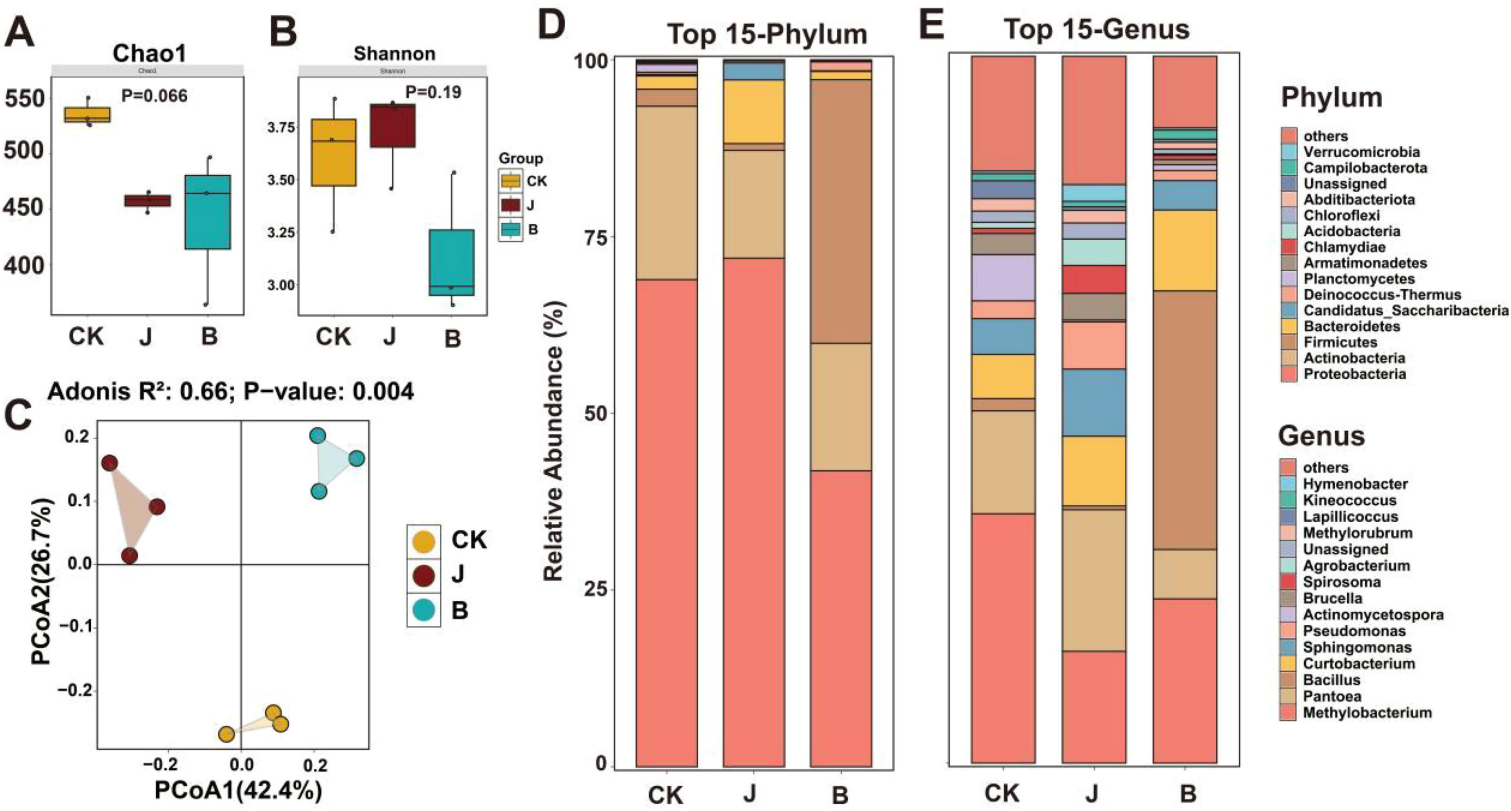
Diversity and composition analysis of microbial communities under different treatments. **(A)** Chao1 index. **(B)** Shannon index. **(C)** Principal coordinate analysis (PCoA). **(D)** relative abundance of microbial communities at the phylum level. **(E)** relative abundance of microbial communities at the genus level.

To evaluate the impact of different treatments on the rice phyllosphere microbiome, this study analyzed the diversity and structural differences of the bacterial communities on the rice leaf surface, focusing on the effects of B, J, and CK treatments. The results showed that different treatments significantly affected the diversity and composition of the rice microbiome. In terms of alpha diversity, the Chao1 index indicated that the B and J treatments had lower values than the CK treatment, but the difference was not statistically significant (*p* = 0.066) (Figure 5A). The Shannon index showed that the community evenness in the B treatment was lower than in the J and CK treatments, but the difference was not significant (*p* = 0.19) (Figure 5B). In terms of beta diversity, the PCoA analysis revealed clear separation of the microbial community structures among the three treatments, indicating significant differences in community structure *(p* = 0.004). PCoA1 and PCoA2 explained 42.4 and 26.7% of the variation, respectively (Figure 5C). At the phylum level, the B treatment showed a significant decrease in *Proteobacteria* and *Bacteroidetes* compared to the CK and J treatments, while *Firmicutes* significantly increased (Figure 5D). At the genus level, the B treatment showed a significant decrease in *Pantoea* and *Sphingomonas* compared to the CK and J treatments, while *Curtobacterium* and *Bacillus* significantly increased (Figure 5E).

**FIGURE 6:**
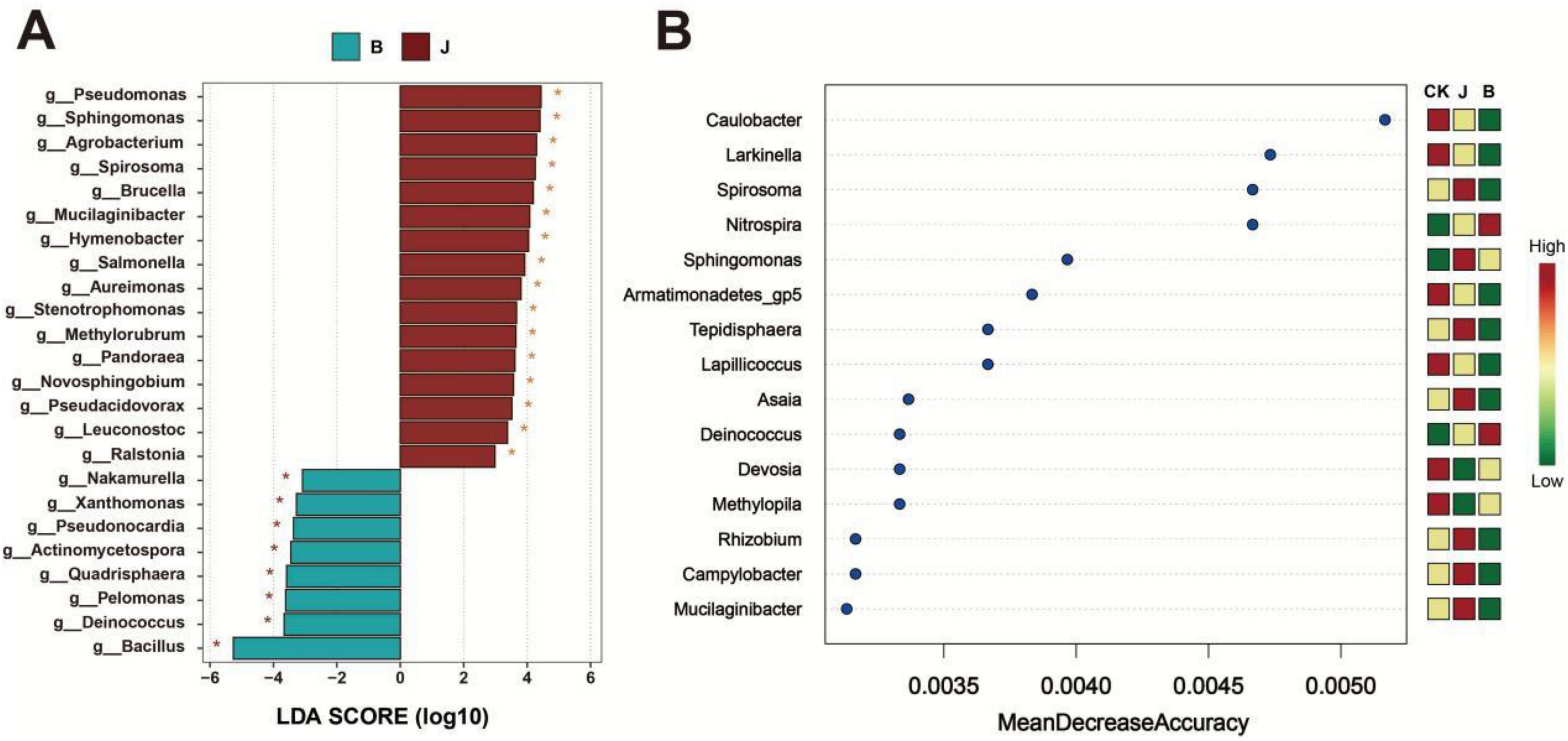
Microbial community analysis in rice under different treatments. **(A)** Linear discriminant analysis (LDA) score plot showing significant microbial differences between the B and J treatments. **(B)** Random forest analysis displaying the importance ranking of microbial genera, based on the mean decrease accuracy (MDA) value.

The analysis of the microbial community at the genus level showed significant differences between the B and J treatments. LEfSe analysis (Figure 6A) indicated that there were distinct differences in the abundance of multiple microbial genera between the B and J treatments. Specifically, the J treatment exhibited higher LDA scores in genera such as *Pseudomonas*, *Sphingomonas*, and *Agrobacterium*, while the B treatment showed higher scores in *Bacillus*, *Deinococcus*, and *Pelomonas*, with the differences being statistically significant (*p* < 0.05). Random forest analysis (Figure 6B) revealed differences in the genus-level classification accuracy among the B, J, and CK treatments. Several genera with higher MDA scores, such as *Caulobacter*, *Larkinella*, *Spirosoma*, and *Nitrospira*, contributed significantly to distinguishing the two treatments. *Caulobacter* and *Larkinella* had higher abundances in the CK treatment, *Spirosoma* was more abundant in the J treatment, and *Nitrospira* had the highest abundance in the B treatment.

### 3.6 Effects of different treatments on phyllosphere microbial community-related pathways in rice

**FIGURE 7:**
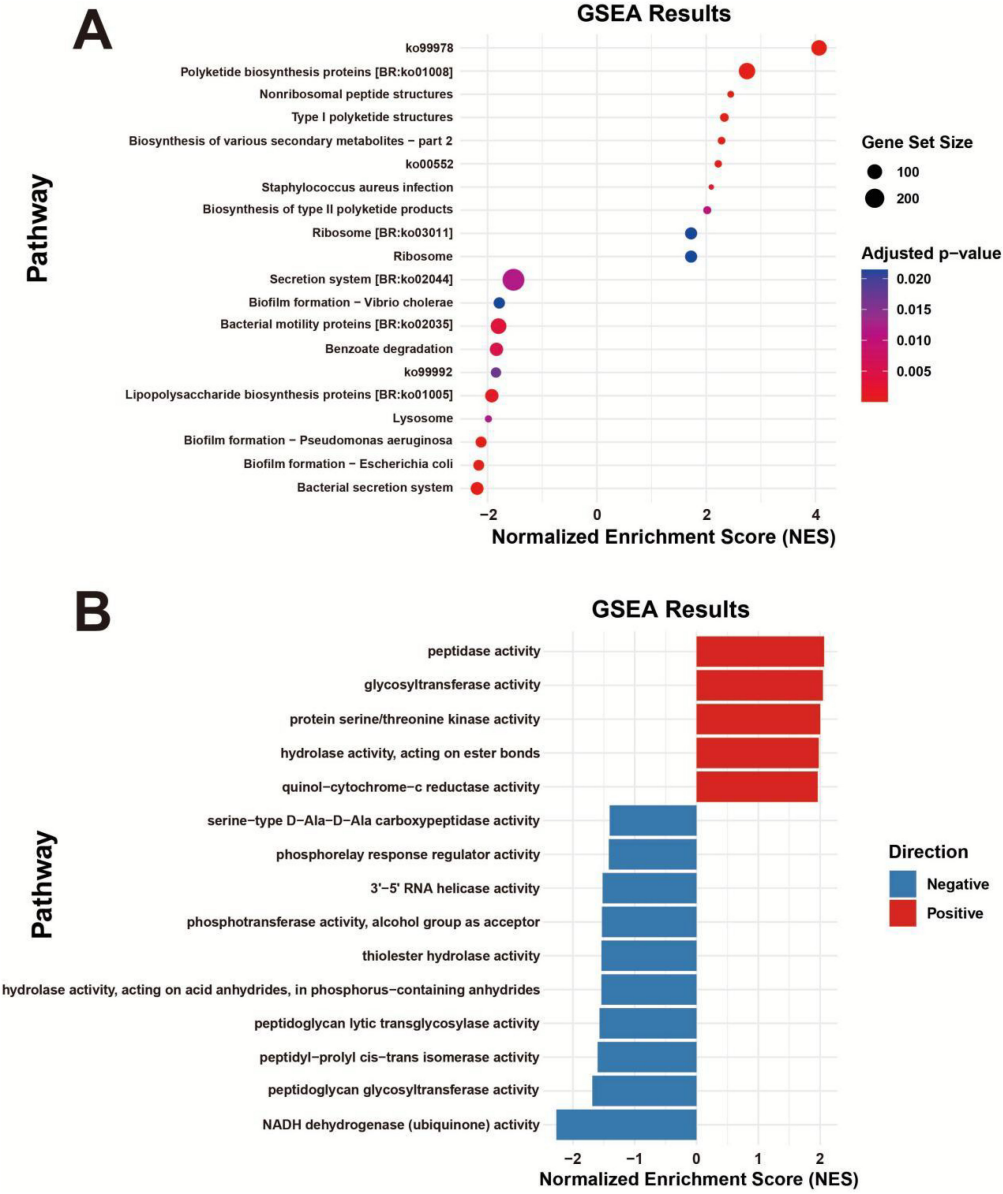
GSEA pathway enrichment analysis. **(A)** KEGG pathway enrichment analysis, sorted by normalized enrichment score (NES), where the dot size represents the gene set size, and the color indicates the adjusted *p*-value. **(B)** GO pathway enrichment analysis, with blue indicating negative enrichment and red indicating positive enrichment. B represents the treatment with *Bacillus velezensis* BER1, and J represents the validamycin treatment.

To gain a deeper understanding of the impact of different treatments on the functionality of the rice phyllosphere microbial community, this experiment explored the differences in relevant pathways and enzyme activities between the B and J treatments through gene set enrichment analysis. The results revealed significant differences in the microbial community-related pathways and enzyme activities between the B and J treatments. KEGG functional prediction (Figure 7A) showed that the B treatment primarily enriched pathways closely related to microbial and plant secondary metabolism, such as Polyketide biosynthesis proteins, Nonribosomal peptide structures, Type I polyketide structures, and Biosynthesis of various secondary metabolites—part 2. In contrast, the J treatment primarily enriched pathways related to bacterial survival adaptation and metabolic processes, including secretion system, bacterial motility proteins, and benzoate degradation. GO functional prediction (Figure 7B) revealed that the B treatment was enriched in peptidase activity, glycosyltransferase activity, protein serine/threonine kinase activity, hydrolase activity, acting on ester bonds, and quinol-cytochrome-c reductase activity, while the J treatment showed more significant enzyme activities in certain hydrolases and NADH dehydrogenases.

AntiSMASH analysis combined with PICRUSt2 KO annotations revealed the functional assignments of core biosynthetic genes in several secondary metabolite gene clusters (Table 4). In cluster1 (transAT-PKS-like type), the genes *pksG* (K15311), *pksH* (K15312), *pksI* (K15313), *pksC* (K15327), and *acpK* (K15337) were identified. The KOs corresponding to these genes are associated with core enzyme functions in the polyketide natural product synthesis process, consistent with the product difficidin predicted by antiSMASH. In cluster4, the two genes *pksG* (K15311) and *pksI* (K15313) were also annotated as KOs related to secondary metabolite biosynthesis. AntiSMASH predicted that this cluster might produce the bioactive natural product *fengycin*. KO description information indicates that these KOs correspond to key enzymes in polyketide biosynthesis.

**TABLE 4:** Integrated annotation of secondary metabolite biosynthetic gene clusters and KEGG ortholog (KO) functions based on antiSMASH and PICRUSt2.

Cluster ID	KO ID	Gene name	Gene ID	Type	Predicted product	KO description
Cluster1	K15311	*pksG*	Gene0146	transAT-PKS-like	Difficidin	Polyketide biosynthesis 3-hydroxy-3-methylglutaryl-CoA synthase-like enzyme *PksG*
Cluster4	K15311	*pksG*	Gene2267	NRPS	Fengycin	
Cluster1	K15312	*pksH*	Gene0145	transAT-PKS-like	Difficidin	Polyketide biosynthesis enoyl-CoA hydratase *PksH*
Cluster1	K15313	*pksI*	Gene0144	transAT-PKS-like	Difficidin	Polyketide biosynthesis enoyl-CoA hydratase *PksI*
Cluster4	K15313	*pksI*	Gene2268	NRPS	Fengycin	
Cluster1	K15327	*pksC*	Gene0150	transAT-PKS-like	Difficidin	Polyketide biosynthesis malonyl-CoA-[acyl-carrier-protein] transacylase
Cluster1	K15337	*acpK*	Gene0147	transAT-PKS-like	Difficidin	Polyketide biosynthesis acyl carrier protein

### 3.7 Phylogenetic phenotypic prediction of rice phyllosphere microbial communities based on BugBase

**FIGURE 8:**
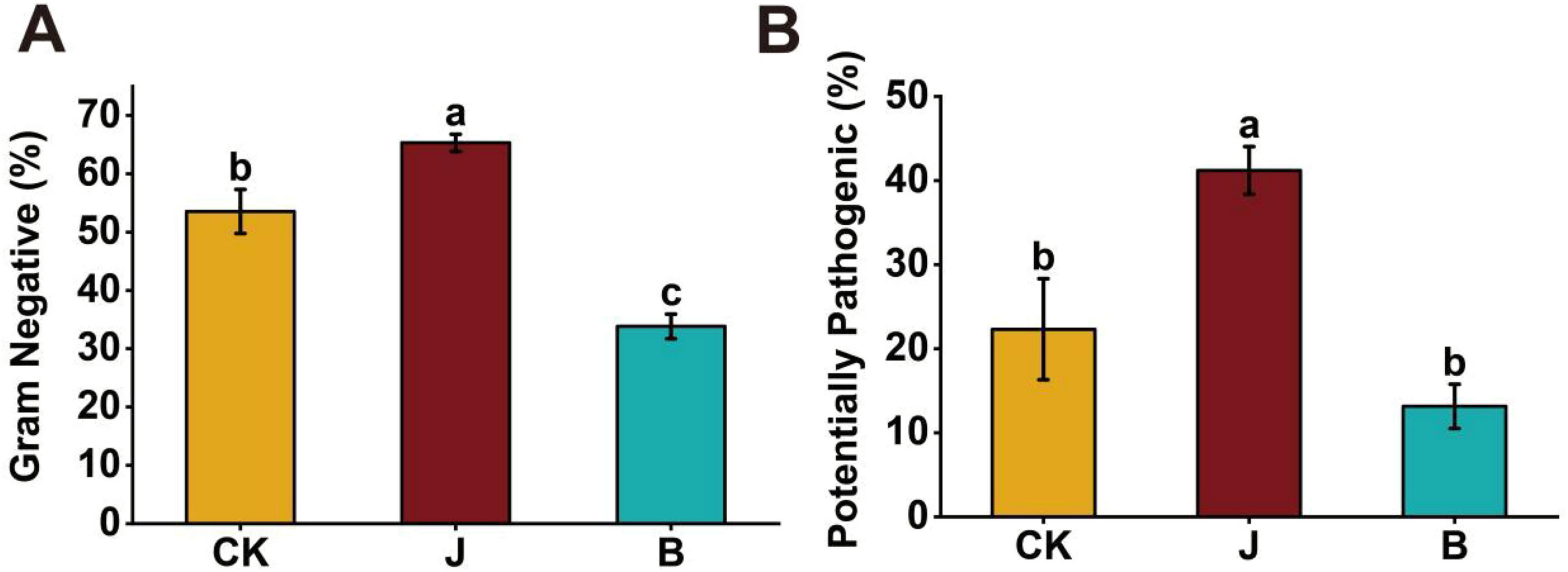
Proportion of gram-negative bacteria and potential pathogens in rice microbial communities based on BugBase phenotypic prediction. **(A)** Proportion of Gram-negative bacteria in rice leaves based on BugBase prediction, with differences between treatments indicated by letters (*p* < 0.05). **(B)** Proportion of potential pathogenic bacteria in rice leaves based on BugBase prediction, with differences between treatments indicated by letters (*p* < 0.05).

To assess the impact of different treatments on the rice microbiome, BugBase-based phenotypic prediction was used to analyze the proportion of Gram-negative bacteria and potential pathogenic bacteria. The results showed significant differences in the proportions of Gram-negative bacteria and potential pathogens in the rice microbial community between the B and J treatments. The proportion of Gram-negative bacteria in the J treatment (62.29%) was significantly higher than in the CK treatment (53.53%) and B treatment (33.82%) (Figure 8A). The proportion of potential pathogenic bacteria in the J treatment was significantly higher than in the CK and B treatments, at 41.19%, followed by CK at 22.31%, with B treatment having the lowest at 13.14% (Figure 8B).

## 4 Discussion

### 4.1 Direct antagonism mediated by BER1 and synergistic enhancement of host systemic immunity in disease resistance mechanisms

In this study, pot experiments with rice plants showed that *Bacillus velezensis* BER1 significantly reduced the disease severity of rice sheath blight, with its overall efficacy surpassing the commonly used chemical treatment, J treatment (Figure 3), demonstrating biocontrol potential comparable to chemical control. This finding is consistent with numerous reports on *Bacillus velezensis* as an efficient biocontrol agent, confirming the strain’s advantages in disease resistance mechanisms. Whole-genome antiSMASH analysis revealed that BER1 contains multiple biosynthetic gene clusters closely related to secondary metabolite synthesis, including lipopeptides, polyketides, and small peptide antibiotics. Lipopeptides such as surfactin and fengycin are synthesized by nonribosomal peptide synthetases (NRPS) and exhibit significant antifungal activity (Table 2; Ongena and Jacques, 2008; Raaijmakers et al., 2010). Surfactin is not only a potent biosurfactant capable of disrupting the integrity of pathogen cell membranes but also promotes colonization of the strain on the host surface and induces plant immune responses (Crouzet et al., 2020; Jasińska et al., 2026). Fengycin is particularly effective against filamentous fungi and is one of the major chemical inhibitors of fungal pathogens in lipopeptides (Vanittanakom et al., 1986; Deleu et al., 2008). Polyketide antimicrobial compounds such as bacillaene and macrolactin are synthesized by polyketide synthases (PKS) and primarily exert antibacterial effects by interfering with bacterial protein synthesis or other key metabolic processes. Small peptide antibiotics like bacilysin and subtilin inhibit pathogen metabolism through interactions with target cell walls, displaying antibacterial activity against certain bacteria and fungi (Patel et al., 1995; Yuan et al., 2016; Caulier et al., 2019).

*In vitro* experiments showed that *Bacillus velezensis* BER1 exhibited high extracellular enzyme activity, including glucanase, chitinase, protease, and a strong siderophore release capacity (Table 1). Glucanase and chitinase inhibit fungal hyphal extension and spore germination by degrading β-1,3-glucan and chitin in fungal cell walls (Adams, 2004). Protease degrades key structural proteins of pathogens, inhibiting their growth. Siderophores inhibit pathogen growth by competing with Feł^+^ and reducing the available iron sources (Han et al., 2017; Xie et al., 2024). More importantly, these extracellular enzymes and siderophores not only exhibit direct antifungal effects but also serve as signal triggers for inducing systemic resistance (ISR). For example, the fragments generated by hydrolytic enzymes degrading pathogen components can act as PAMPs to activate the host immune system (Trouvelot et al., 2014). Siderophores, while competing for iron resources, also indirectly stimulate host iron stress-related signals, thereby enhancing the sensitivity of the host’s immune response (Pandey, 2023).

Through the analysis of rice immune responses, this study further confirmed that *Bacillus velezensis* BER1 can trigger host immunity (Figure 4), a mechanism involving the coordinated regulation of multiple immune signaling pathways. *Bacillus velezensis* BER1 not only competes directly with pathogens and produces disease-active compounds, but also acts as an immune inducer to trigger host ISR (induced systemic resistance) and SAR (systemic acquired resistance) pathways. Both ISR and SAR function through complex plant hormone signaling networks, with salicylic acid (SA) and jasmonic acid (JA) signaling pathways playing crucial roles in regulating disease resistance. SAR primarily depends on the SA signaling pathway and the expression of related downstream defense genes, while ISR generally involves JA/ethylene and other signaling pathways in a complex regulatory network. Both pathways can synergistically or interactively influence the host immune response. In this study, the upregulation of NH1 suggested the activation of the SA-dependent immune pathway. NH1, a core regulatory factor in the NPR1 family, serves as a central node in the joint regulatory network of SAR and ISR, becoming activated in response to SA signals and initiating the expression of multiple downstream disease resistance genes (such as *PR1a* and *PR10*). Similar studies have shown that metabolites produced by probiotics can induce the expression of host PR genes, increase SA levels, and enhance disease resistance (Li et al., 2022). Meanwhile, the upregulation of *LOX* and *AOS*_2_ indicated that the JA pathway was simultaneously activated. The JA pathway plays a key role in plant responses to pathogen invasion, inducing the expression of JA-related resistance genes and defense responses, and serving as a core regulatory mechanism in ISR. This finding is consistent with previous reports that *Bacillus* biocontrol strains induce elevated JA levels and enhance host immunity (Niu et al., 2016; Dimopoulou et al., 2019). At the level of immune output, the activity of defense-related enzymes, such as POD and PAL, was significantly increased, in line with the dynamic changes in immune signaling pathway activation (Table 3). POD reinforces the cell wall, generates reactive oxygen species (ROS), and acts as a PR-9 enzyme to exert antimicrobial effects; increased PAL activity promotes the accumulation of antimicrobial secondary metabolites, such as lignin, enhancing structural defenses. In addition, the moderately increased CAT activity reflects the balanced regulation of ROS signaling. While ROS contribute to immune responses and cell wall reinforcement, excessive accumulation may cause oxidative damage. Therefore, ROS-clearing enzymes such as CAT are critical for maintaining immune effectiveness and host homeostasis. Previous studies have shown that the coordination of ROS generation and clearance is essential for effective immune responses (Rogers and Munné-Bosch, 2016; Lee et al., 2020; Sood, 2025).

In summary, the mechanism by which *Bacillus velezensis* BER1 triggers rice immunity can be summarized as a synergistic interaction of two aspects: on one hand, *Bacillus velezensis* BER1 effectively inhibits pathogen growth through its secondary metabolites and extracellular enzymes; on the other hand, its phyllosphere colonization activates the host’s systemic resistance network, including the SA-dependent systemic acquired resistance (SAR) and JA/ET-related induced systemic resistance (ISR) signaling pathways. These two types of induced resistance collectively enhance the expression of downstream defense genes and enzyme activities, strengthening the immune output and defense barriers of rice. Moreover, moderate ROS regulation ensures a balance between enhanced immunity and host homeostasis. These mechanisms align with the hormone regulation and immune enhancement patterns described in plant-induced systemic resistance and systemic acquired resistance, indicating that the immune state triggered by probiotics provides an effective strategy to enhance crop disease resistance.

### 4.2 BER1 enhances plant disease resistance by dominant microbial colonization and secondary metabolic pathway enrichment in the phyllosphere microbiom

This study revealed the significant impact of *Bacillus velezensis* BER1 treatment on the microbial community structure and potential functional output at the phyllosphere microbial community level. The phyllosphere, as an important microecosystem of the plant aerial parts, consists of a variety of bacteria, fungi, and other microorganisms that not only participate in resource competition but also influence plant health through various interactions (Lindow and Brandl, 2003). *Bacillus velezensis* BER1 treatment significantly increased the abundance of *Firmicutes* (primarily *Bacillus*) while reducing the relative abundance of certain potential pathogenic bacteria (e.g., some *Proteobacteria* and related genera) (Figure 5). This dominant colonization not only indicates that the dominant strains occupy ecological niches in the nutrient-limited phyllosphere environment but also suggests that by occupying space and resources, the opportunity for pathogen colonization is reduced. On one hand, *Bacillus* species in the community often synthesize antimicrobial lipopeptides and polyketides (Alizadeh, 2025). On the other hand, the community structural adjustment triggered by BER1 also implies that the phyllosphere microbiome is evolving toward a functional direction with stronger resource competition, metabolic activity, and host interaction ability, thereby enhancing the disease resistance of the community (Du et al., 2025). In the random forest analysis, genera such as *Caulobacter*, *Larkinella*, *Spirosoma*, and *Nitrospira* showed higher MDA values (Figure 6), indicating that these genera are the most important features for distinguishing the three treatments. From the treatment abundance pattern, *Nitrospira* had the highest abundance in the B treatment. As an obligate nitrifying bacterium, *Nitrospira* can oxidize nitrite to nitrate and is a key chemotrophic member in the nitrogen cycle, widely present in natural environments and various microhabitats (Daims et al., 2015; Ushiki et al., 2018). In contrast, *Spirosoma* exhibited higher abundance in the J treatment, possibly due to the changes in carbon sources and microenvironmental nutrient dynamics induced by the chemical treatment (Lail et al., 2010). *Caulobacter* and *Larkinella* had relatively higher abundance in the CK treatment, which may reflect the native homeostasis of the phyllosphere microbiome in the absence of intervention.

Functional prediction analysis showed that after *Bacillus velezensis* BER1 treatment, not only did the community composition change, but the overall potential functional profile of the phyllosphere microbiome significantly enriched KEGG pathways related to secondary metabolite biosynthesis, particularly natural product synthesis pathways such as polyketide biosynthesis (Figure 7). These secondary metabolite biosynthesis pathways typically involve the production of antimicrobial polyketide compounds, a functional characteristic closely related to microbial-mediated pathogen suppression and the maintenance of systemic resistance. Previous studies have indicated that secondary metabolite gene clusters (BGCs) enriched in plant-associated microbial communities can encode various bioactive natural products, including nonribosomal peptides (NRPs), polyketides, siderophores, and other small antimicrobial molecules, which can provide direct disease resistance effects or indirectly enhance the host immune state (Ziemert et al., 2016; Dror et al., 2023). Combining the whole-genome analysis of BER1 with the KO annotations from PICRUSt2, this study identified core enzyme functions associated with polyketide biosynthesis in several core secondary metabolite gene clusters (Table 4).

In cluster1, multiple genes were identified that are essential core enzymes in the biosynthesis of polyketide natural products and are consistent with the biosynthetic pathway of *difficidin*, predicted by antiSMASH. However, given the moderate similarity (53%), this annotation remains tentative and requires further validation, despite PICRUSt2 prediction. Difficidin is a macrolide-like polyketide antibiotic produced via the Type I PKS pathway, with reported broad-spectrum antimicrobial activity (Chen et al., 2006; Caulier et al., 2019). *Difficidin* rapidly blocks protein synthesis in pathogens, significantly inhibiting protein synthesis (Chen et al., 2009). Additionally, difficidin has been reported to inhibit the growth of plant pathogens, including *Xanthomonas* and *Pseudomonas* species (Fazle Rabbee and Baek, 2023). In cluster4, genes identified pointed to the potential secondary metabolic pathway of the bioactive natural product *fengycin*, whose basic antifungal mechanism is mainly derived from the direct disruption of fungal cell membranes and cell walls and the interference with fungal intracellular homeostasis. On one hand, the amphipathic structure of *fengycin* allows it to bind tightly to the fungal extracellular membrane lipids and cell wall components, disrupting membrane integrity and altering membrane permeability, leading to ion leakage and cell content leakage, thereby directly inhibiting hyphal growth and extension (Botcazon et al., 2024). On the other hand, *fengycin* is frequently observed to induce cell membrane rupture and organelle dysfunction, disturb metabolism within the fungus, and even trigger programmed cell death or autophagic responses (Zakharova et al., 2019; Chen et al., 2025).

BugBase phenotypic prediction showed that the J treatment had a higher relative abundance of Gram-negative and potentially pathogenic bacteria (Figure 8). Following fungicide application, Gram-negative bacteria may increase, possibly due to shifts in microbial community structure (Legein et al., 2020). In contrast, the introduction of *Bacillus velezensis* BER1 stabilizes ecological competition and releases disease-resistant metabolites, limiting the expansion of resistant bacteria and pathogens, thereby maintaining the microecological balance. This suggests that the mechanisms by which chemical agents and biocontrol bacteria influence the phyllosphere microbiome differ; the former may promote pathogen proliferation, whereas biocontrol bacteria help reduce this risk (Hernandez et al., 2021; Negi et al., 2026).

In summary, the BER1 treatment suppresses pathogens by establishing a “disease-suppressive microbial ecosystem” in the phyllosphere microbiome. Following the introduction of *Bacillus velezensis* BER1, there was a significant enrichment of *Bacillus* species, forming a dominant population that reduced the colonization opportunities of opportunistic pathogens. The treatment enhanced defense pathways related to secondary metabolite biosynthesis and, through stable ecological competition and metabolic outputs, maintained ecological balance, thereby lowering the risk of potential pathogenic phenotypes. This mechanism not only relies on the disease resistance of the probiotics but also involves the comprehensive action of the entire community functional network, reflecting the characteristics of a natural disease-suppressive microbiome.

## 5 Conclusion

This study selected and identified *Bacillus velezensis* BER1 from 10 biocontrol strains, revealing its mechanisms for controlling rice sheath blight from genomic, plant immune response, and microecological perspectives. The BER1 treatment significantly reduced disease severity, achieving a control efficacy of 60.0%, which was superior to chemical treatments. Genomic analysis indicated that BER1 carries secondary metabolite biosynthetic gene clusters, such as fengycin, bacillaene, and macrolactin, demonstrating broad-spectrum antifungal and antibacterial activity. In rice immune responses, BER1 significantly induced the expression of immune-related genes such as *AOS*_2_ and *PR1a*, and enhanced the activity of defense enzymes like POD and PAL. 16S rRNA sequencing results showed that BER1 significantly increased the relative abundance of *Bacillus* species while inhibiting the proportion of potential pathogens. Functional prediction also revealed enhanced pathways related to polyketide synthesis, suggesting that BER1 promoted disease resistance functions in the phyllosphere microbial community through microecological reshaping. This multi-level synergistic mechanism confirms the potential of BER1 as an effective biocontrol agent, providing data support and theoretical basis for plant disease management strategies based on microecological regulation, and contributing to the development of green rice sheath blight control technologies.

## Data Availability

The sequencing data have now been updated and are publicly available in the NCBI Sequence Read Archive (SRA) under the BioProject accession number PRJNA1441215. The data can be accessed at: https://www.ncbi.nlm.nih.gov/sra/?term=PRJNA1441215.

## References

[B1] Adams D. J. (2004). Fungal cell wall chitinases and glucanases. *Microbiology* 150 2029–2035. 10.1099/mic.0.26980-0 15256547

[B2] Ali M. A. Ahmed T. Ibrahim E. Rizwan M. Chong K. P. Yong J. W. H. (2024). A review on mechanisms and prospects of endophytic bacteria in biocontrol of plant pathogenic fungi and their plant growth-promoting activities. *Heliyon* 10:e31573. 10.1016/j.heliyon.2024.e31573 38841467 PMC11152693

[B3] Alizadeh M. (2025). Unveiling the phyllosphere microbiome: Guardians of tree health and environmental resilience. *Physiol. Mol. Plant Pathol.* 140:102914. 10.1016/j.pmpp.2025.102914

[B4] Botcazon C. Ramos-Martín F. Rodríguez-Moraga N. Bergia T. Acket S. Sarazin C. et al. (2024). Rhamnolipids and fengycins interact differently with biomimetic lipid membrane models of *Botrytis cinerea* and *Sclerotinia sclerotiorum*: Lipidomics profiles and biophysical studies. *Biophys. Chem.* 314:107305. 10.1016/j.bpc.2024.107305 39154582

[B5] Caulier S. Nannan C. Gillis A. Licciardi F. Bragard C. Mahillon J. (2019). Overview of the antimicrobial compounds produced by members of the *Bacillus subtilis* group. *Front. Microbiol.* 10:302. 10.3389/fmicb.2019.00302 30873135 PMC6401651

[B6] Chen J. Xuan Y. Yi J. Xiao G. Yuan D. P. Li D. (2023). Progress in rice sheath blight resistance research. *Front. Plant Sci.* 14:1141697. 10.3389/fpls.2023.1141697 37035075 PMC10080073

[B7] Chen M. Wang H. Zhang C. Zhang Y. Sun H. Yu L. et al. (2025). Recent advances in antimicrobial lipopeptide fengycin secreted by *Bacillus*: Structure, biosynthesis, antifungal mechanisms, and potential application in food preservation. *Food Chem.* 489:144937. 10.1016/j.foodchem.2025.144937 40460478

[B8] Chen X. H. Scholz R. Borriss M. Junge H. Mögel G. Kunz S. et al. (2009). Difficidin and bacilysin produced by plant-associated *Bacillus amyloliquefaciens* are efficient in controlling fire blight disease. *J. Biotechnol.* 140 38–44. 10.1016/j.jbiotec.2008.10.015 19061923

[B9] Chen X. Vater J. Piel J. Franke P. Scholz R. Schneider K. et al. (2006). Structural and functional characterization of three polyketide synthase gene clusters in *Bacillus amyloliquefaciens* FZB 42. *J. Bacteriol.* 188 4024–4036. 10.1128/jb.00052-06 16707694 PMC1482889

[B10] Chen Z. Wang Z. Xu W. (2024). *Bacillus velezensis* WB induces systemic resistance in watermelon against Fusarium wilt. *Pest Manag. Sci.* 80 1423–1434. 10.1002/ps.7873 37939121

[B11] Crouzet J. Arguelles Arias A. Dhondt-Cordelier S. Cordelier S. Pršić J. Hoff G. et al. (2020). Biosurfactants in plant protection against diseases: Rhamnolipids and lipopeptides case study. *Front. Bioeng. Biotechnol.* 8:1014. 10.3389/fbioe.2020.01014 33015005 PMC7505919

[B12] Daims H. Lebedeva E. V. Pjevac P. Han P. Herbold C. Albertsen M. et al. (2015). Complete nitrification by *Nitrospira bacteria*. *Nature* 528 504–509. 10.1038/nature16461 26610024 PMC5152751

[B13] Deleu M. Paquot M. Nylander T. (2008). Effect of fengycin, a lipopeptide produced by *Bacillus subtilis*, on model biomembranes. *Biophys. J.* 94 2667–2679. 10.1529/biophysj.107.114090 18178659 PMC2267117

[B14] Dimopoulou A. Theologidis I. Liebmann B. Kalantidis K. Vassilakos N. Skandalis N. (2019). *Bacillus amyloliquefaciens* MBI600 differentially induces tomato defense signaling pathways depending on plant part and dose of application. *Sci. Rep.* 9:19120. 10.1038/s41598-019-55645-2 31836790 PMC6910970

[B15] Dobrzyński J. Jakubowska Z. (2025). *Pseudomonas* protegens as a biocontrol agent against phytopathogenic fungi - mini review. *World J. Microbiol. Biotechnol.* 41:428. 10.1007/s11274-025-04643-w 41174125 PMC12578757

[B16] Dror B. Jurkevitch E. Cytryn E. (2023). Global-scale analysis reveals distinct patterns of non-ribosomal peptide and polyketide synthase encoding genes in root and soil bacterial communities. *Soil Ecol. Lett.* 5 38–45. 10.1007/s42832-022-0146-2

[B17] Du Y. Han X. Tsuda K. (2025). Microbiome-mediated plant disease resistance: Recent advances and future directions. *J. Gen. Plant Pathol.* 91 1–17. 10.1007/s10327-024-01204-1

[B18] Fazle Rabbee M. Baek K. (2020). Antimicrobial activities of lipopeptides and polyketides of *Bacillus velezensis* for agricultural applications. *Molecules* 25:4973. 10.3390/molecules25214973 33121115 PMC7662345

[B19] Fazle Rabbee M. Baek K. (2023). Detection of antagonistic compounds synthesized by *Bacillus velezensis* against *Xanthomonas citri* subsp. citri by metabolome and RNA sequencing. *Microorganisms* 11:1523. 10.3390/microorganisms11061523 37375024 PMC10301053

[B20] Han Z. Kautto L. Nevalainen H. (2017). Secretion of proteases by an opportunistic fungal pathogen *Scedosporium aurantiacum*. *PLoS One* 12:e0169403. 10.1371/journal.pone.0169403 28060882 PMC5218550

[B21] Hernandez D. J. David A. S. Menges E. S. Searcy C. A. Afkhami M. E. (2021). Environmental stress destabilizes microbial networks. *ISME J.* 15 1722–1734. 10.1038/s41396-020-00882-x 33452480 PMC8163744

[B22] Jasińska A. Walaszczyk A. Bernat P. Trzcinski P. Górnik K. Sas-Paszt L. et al. (2026). Biosurfactant-producing *Bacillus spp*. *Suppress Fusarium* via fungal membrane disruption and promote cucumber growth. *Sci. Rep.* 16:9460. 10.1038/s41598-026-40391-z 41703286 PMC13004851

[B23] Kurniawati M. Purkan P. Sumarsih S. Baktir A. (2020). A novel 1,3-β-glucanase gene from the metagenomic expression library of Achatina fulica’s digestive gland. *Iran. J. Pharmaceut. Res. IJPR* 19 483–493. 10.22037/ijpr.2020.1101172 33680046 PMC7757977

[B24] Lail K. Sikorski J. Saunders E. Lapidus A. Glavina Del, Rio T. et al. (2010). Complete genome sequence of *Spirosoma linguale* type strain (1t). *Standards Genom. Sci.* 2 176–185. 10.4056/sigs.741334 21304700 PMC3035282

[B25] Lee D. Lal N. K. Lin Z. D. Ma S. Liu J. Castro B. et al. (2020). Regulation of reactive oxygen species during plant immunity through phosphorylation and ubiquitination of RBOHD. *Nat. Commun.* 11:1838. 10.1038/s41467-020-15601-5 32296066 PMC7160206

[B26] Legein M. Smets W. Vandenheuvel D. Eilers T. Muyshondt B. Prinsen E. et al. (2020). Modes of action of microbial biocontrol in the phyllosphere. *Front. Microbiol.* 11:1619. 10.3389/fmicb.2020.01619 32760378 PMC7372246

[B27] Li D. Li S. Wei S. Sun W. (2021). Strategies to manage rice sheath blight: Lessons from interactions between rice and *Rhizoctonia solani*. *Rice* 14:21. 10.1186/s12284-021-00466-z 33630178 PMC7907341

[B28] Li Q. Hou Z. Zhou D. Jia M. Lu S. Yu J. (2022). A plant growth-promoting bacteria *Priestia megaterium* JR48 induces plant resistance to the crucifer black rot via a salicylic acid-dependent signaling pathway. *Front. Plant Sci.* 13:1046181. 10.3389/fpls.2022.1046181 36438094 PMC9684715

[B29] Lindow S. E. Brandl M. T. (2003). Microbiology of the phyllosphere. *Appl. Environ. Microbiol.* 69 1875–1883. 10.1128/AEM.69.4.1875-1883.2003 12676659 PMC154815

[B30] Liu X. Bai X. Qian Q. Wang X. Chen M. Chu C. (2005). OsWRKY03, a rice transcriptional activator that functions in defense signaling pathway upstream of OsNPR1. *Cell Res.* 15 593–603. 10.1038/sj.cr.7290329 16117849

[B31] Montejano-Ramírez V. Ávila Oviedo J. Campos Mendoza F. Valencia-Cantero E. (2024). Microbial volatile organic compounds: Insights into plant defense. *Plants* 13:13152013. 10.3390/plants13152013 39124131 PMC11314544

[B32] Mukherjee P. K. Mendoza-Mendoza A. Zeilinger S. Horwitz B. A. (2022). Mycoparasitism as a mechanism of Trichoderma-mediated suppression of plant diseases. *Fungal Biol. Rev.* 39 15–33. 10.1016/j.fbr.2021.11.004

[B33] Nazari M. T. Schommer V. A. Braun J. C. A. Dos Santos L. F. Lopes S. T. Simon V. et al. (2023). Using *Streptomyces spp*. As plant growth promoters and biocontrol agents. *Rhizosphere* 27:100741. 10.1016/j.rhisph.2023.100741

[B34] Negi R. Sharma B. Jyothi R. S. Gupta A. Parastesh F. Kaur T. et al. (2026). Phyllosphere microbiome: Exploring the unexplored frontiers for precision agricultural and environmental sustainability. *World J. Microbiol. Biotechnol.* 42:50. 10.1007/s11274-026-04788-2 41549150

[B35] Nguyen H. Pham T. Nguyen P. Dinh N. Le M. Nguyen T. et al. (2025). Microbial biocontrol in agriculture: From mechanistic understanding to field application. *Discover Plants* 2:334. 10.1007/s44372-025-00421-y

[B36] Niu D. Xia J. Jiang C. Qi B. Ling X. Lin S. et al. (2016). Bacillus cereus AR156 primes induced systemic resistance by suppressing mir825/825* and activating defense-related genes in arabidopsis. *J. Integr. Plant Biol.* 58 426–439. 10.1111/jipb.12446 26526683 PMC5028193

[B37] Ongena M. Jacques P. (2008). Bacillus lipopeptides: Versatile weapons for plant disease biocontrol. *Trends Microbiol.* 16 115–125. 10.1016/j.tim.2007.12.009 18289856

[B38] Pandey S. (2023). The role of iron in phytopathogenic microbe–plant interactions: Insights into virulence and host immune response. *Plants* 12:3173. 10.3390/plants12173173 37687419 PMC10563075

[B39] Patel P. S. Huang S. Fisher S. Pirnik D. Aklonis C. Dean L. et al. (1995). Bacillaene, a novel inhibitor of procaryotic protein synthesis produced by *Bacillus subtilis*: Production, taxonomy, isolation, physico-chemical characterization and biological activity. *J. Antibiot.* 48 997–1003. 10.7164/antibiotics.48.997 7592068

[B40] Pathak V. M. Verma V. K. Rawat B. S. Kaur B. Babu N. Sharma A. et al. (2022). Current status of pesticide effects on environment, human health and it’s eco-friendly management as bioremediation: A comprehensive review. *Front. Microbiol.* 13:962619. 10.3389/fmicb.2022.962619 36060785 PMC9428564

[B41] Raaijmakers J. M. De Bruijn I. Nybroe O. Ongena M. (2010). Natural functions of lipopeptides from *Bacillus* and *Pseudomonas*: More than surfactants and antibiotics. *FEMS Microbiol. Rev.* 34 1037–1062. 10.1111/j.1574-6976.2010.00221.x 20412310

[B42] Roberts W. K. Selitrennikoff C. P. (1988). Plant and bacterial chitinases differ in antifungal activity. *Microbiology* 134 169–176. 10.1099/00221287-134-1-169 27077644

[B43] Rogers H. Munné-Bosch S. (2016). Production and scavenging of reactive oxygen species and redox signaling during leaf and flower senescence: Similar but different. *Plant Physiol.* 171 1560–1568. 10.1104/pp.16.00163 27208233 PMC4936548

[B44] Sarwar A. Brader G. Corretto E. Aleti G. Abaidullah M. Sessitsch A. et al. (2018). Qualitative analysis of biosurfactants from Bacillus species exhibiting antifungal activity. *PLoS One* 13:e0198107. 10.1371/journal.pone.0198107 29864153 PMC5986119

[B45] Sarwar M. Kremer R. J. (1995). Determination of bacterially derived auxins using a microplate method. *Lett. Appl. Microbiol.* 20 282–285. 10.1111/j.1472-765X.1995.tb00446.x

[B46] Senapati M. Tiwari A. Sharma N. Chandra P. Bashyal B. M. Ellur R. K. et al. (2022). *Rhizoctonia solani* kühn pathophysiology: Status and prospects of sheath blight disease management in rice. *Front. Plant Sci.* 13:881116. 10.3389/fpls.2022.881116 35592572 PMC9111526

[B47] Shin S. H. Lim Y. Lee S. E. Yang N. W. Rhee J. H. (2001). CAS agar diffusion assay for the measurement of siderophores in biological fluids. *J. Microbiol. Methods* 44 89–95. 10.1016/S0167-7012(00)00229-3 11166103

[B48] Sood M. (2025). Reactive oxygen species (ROS): Plant perspectives on oxidative signalling and biotic stress response. *Discover Plants* 2:187. 10.1007/s44372-025-00275-4

[B49] Tao H. Li X. Huo H. Cai Y. Cai A. (2024). Bacillus velezensis y6, a potential and efficient biocontrol agent in control of rice sheath blight caused by *Rhizoctonia solani*. *Microorganisms* 12:1694. 10.3390/microorganisms12081694 39203537 PMC11357648

[B50] Trouvelot S. Héloir M. Poinssot B. Gauthier A. Paris F. Lemaitre-Guillier C. et al. (2014). Carbohydrates in plant immunity and plant protection: Roles and potential application as foliar sprays. *Front. Plant Sci.* 5:592. 10.3389/fpls.2014.00592 25408694 PMC4219568

[B51] Ushiki N. Fujitani H. Shimada Y. Morohoshi T. Sekiguchi Y. Tsuneda S. (2018). Genomic analysis of two phylogenetically distinct nitrospira species reveals their genomic plasticity and functional diversity. *Front. Microbiol.* 8:2637. 10.3389/fmicb.2017.02637 29375506 PMC5767232

[B52] Vanittanakom N. Loeffler W. Koch U. Jung G. (1986). Fengycin–a novel antifungal lipopeptide antibiotic produced by Bacillus subtilis f-29-3. *J. Antibiot.* 39 888–901. 10.7164/antibiotics.39.888 3093430

[B53] Walsh U. Morrissey J. Gara F. O. (2001). *Pseudomonas* for biocontrol of phytopathogens: From functional genomics to commercial exploitation. *Curr. Opin. Biotechnol.* 12 289–295. 10.1016/S0958-1669(00)00212-3 11404107

[B54] Wang G. Ding X. Yuan M. Qiu D. Li X. Xu C. et al. (2006). Dual function of rice OsDR8 gene in disease resistance and thiamine accumulation. *Plant Mol. Biol.* 60 437–449. 10.1007/s11103-005-4770-x 16514565

[B55] Wang L. Li X. Galileya Medison R. Zheng T. Meng X. Sun Z. et al. (2023). Biocontrol efficacy of *Burkholderia pyrrocinia* s17-377 in controlling rice sheath blight. *Biol. Control* 187:105368. 10.1016/j.biocontrol.2023.105368

[B56] Wu L. Li X. Ma L. Borriss R. Wu Z. Gao X. (2018). Acetoin and 2,3-butanediol from *Bacillus amyloliquefaciens* induce stomatal closure in *Arabidopsis thaliana* and *Nicotiana benthamiana*. *J. Exp. Bot.* 69 5625–5635. 10.1093/jxb/ery326 30295868

[B57] Xie B. Wei X. Wan C. Zhao W. Song R. Xin S. et al. (2024). Exploring the biological pathways of siderophores and their multidisciplinary applications: A comprehensive review. *Molecules* 29:2318. 10.3390/molecules29102318 38792179 PMC11123847

[B58] Yao X. Guo H. Zhang K. Zhao M. Ruan J. Chen J. (2023). Trichoderma and its role in biological control of plant fungal and nematode disease. *Front. Microbiol.* 14:1160551. 10.3389/fmicb.2023.1160551 37206337 PMC10189891

[B59] Yu Y. Gui Y. Li Z. Jiang C. Guo J. Niu D. (2022). Induced systemic resistance for improving plant immunity by beneficial microbes. *Plants* 11:386. 10.3390/plants11030386 35161366 PMC8839143

[B60] Yuan J. Zhao M. Rong L. Huang Q. Rensing C. Raza W. et al. (2016). Antibacterial compounds-macrolactin alters the soil bacterial community and abundance of the gene encoding PKS. *Front. Microbiol.* 7:1904. 10.3389/fmicb.2016.01904 27965639 PMC5126139

[B61] Zakharova A. A. Efimova S. S. Malev V. V. Ostroumova O. S. (2019). Fengycin induces ion channels in lipid bilayers mimicking target fungal cell membranes. *Sci. Rep.* 9:16034. 10.1038/s41598-019-52551-5 31690786 PMC6831686

[B62] Zhang N. Wang Z. Shao J. Xu Z. Liu Y. Xun W. et al. (2023). Biocontrol mechanisms of bacillus: Improving the efficiency of green agriculture. *Microb. Biotechnol.* 16 2250–2263. 10.1111/1751-7915.14348 37837627 PMC10686189

[B63] Zhao C. Wang A. Shi Y. Wang L. Liu W. Wang Z. et al. (2008). Identification of defense-related genes in rice responding to challenge by *Rhizoctonia solani*. *Theor. Appl. Genet.* 116 501–516. 10.1007/s00122-007-0686-y 18075727

[B64] Ziemert N. Alanjary M. Weber T. (2016). The evolution of genome mining in microbes – a review. *Nat. Prod. Rep.* 33 988–1005. 10.1039/c6np00025h 27272205

[B65] Zihalirwa Kulimushi P. Argüelles Arias A. Franzil L. Steels S. Ongena M. (2017). Stimulation of fengycin-type antifungal lipopeptides in *Bacillus amyloliquefaciens* in the presence of the maize fungal pathogen rhizomucor variabilis. *Front. Microbiol.* 8:850. 10.3389/fmicb.2017.00850 28555132 PMC5430075

