# Supplementary material for: Genomic and microecological insights into the biocontrol mechanisms of Bacillus velezensis BER1 against rice sheath blight
Source: Front Microbiol. 2026 May 14;17:1836902. doi: 10.3389/fmicb.2026.1836902 (PMC13216488; doi:10.3389/fmicb.2026.1836902)
Supplement: Supplementary file 1 [file Table_1.docx]

Supplementary Materials

**Genomic and Microecological Insights into the Biocontrol Mechanisms of *Bacillus velezensis* BER1 against Rice Sheath Blight**

**Content**

[Table S1. Sequences of primers used for quantitative real-time PCR 1](#_Toc30905)

[Table S2. Classification of Disease Severity in Rice 2](#_Toc16554)

[Figure S1. Antagonistic activity of](#_Toc5306) *[Bacillus velezensis](#_Toc5306)* [BER1 against five plant pathogenic fungi (](#_Toc5306)*[Fusarium oxysporum](#_Toc5306)* [f. sp.](#_Toc5306) *[cubense](#_Toc5306)*[,](#_Toc5306) *[Rhizoctonia](#_Toc5306)**[solani](#_Toc5306)*[,](#_Toc5306) *[Botrytis cinerea](#_Toc5306)*[,](#_Toc5306) *[Fusarium oxysporum](#_Toc5306)* [f. sp.](#_Toc5306) *[niveum](#_Toc5306)*[, and](#_Toc5306) *[Phytophthora](#_Toc5306)**[infestans](#_Toc5306)*[) in dual culture assays after 4 days of incubation at 28°C. 3](#_Toc5306)

# Table S1. Sequences of primers used for quantitative real-time PCR

| Gene | Primer Sequences (5'-3') | |
| --- | --- | --- |
| *NH1* | CACGCCTAAGCCTCGGATA | TCAGTGAGCAGCATCCTGACTAG |
| *PR1a* | CGTCTTCATCACCTGCAACTACTC | CATGCATAAACACGTAGCATAGCA |
| *PR10* | CCCTGCCGAATACGCCTAA | CTCAAACGCCACGAGAATTTG |
| *LOX* | GCATCCCCAACAGCACATC | AATAAAGATTTGGGAGTGACATATTGG |
| *AOS_2_* | CAATACGTGTACTGGTCGAATGG | AAGGTGTCGTACCGGAGGAA |
| *Actin* | TGTATGCCAGTGGTCGTACC | CCAGCAAGGTCGAGACGAA |

# Table S2. Classification of Disease Severity in Rice

| Grade | Disease Severity Criteria |
| --- | --- |
| 0 | No disease observed on the entire plant |
| 1 | Disease observed on the basal leaf sheaths |
| 2 | Disease observed on leaf sheaths or leaves below the third leaf (counting from the top) |
| 3 | Disease observed on leaf sheaths or leaves below the second leaf |
| 4 | Disease observed on the top leaf sheaths or top leaves |
| 5 | The entire plant is diseased and dead |


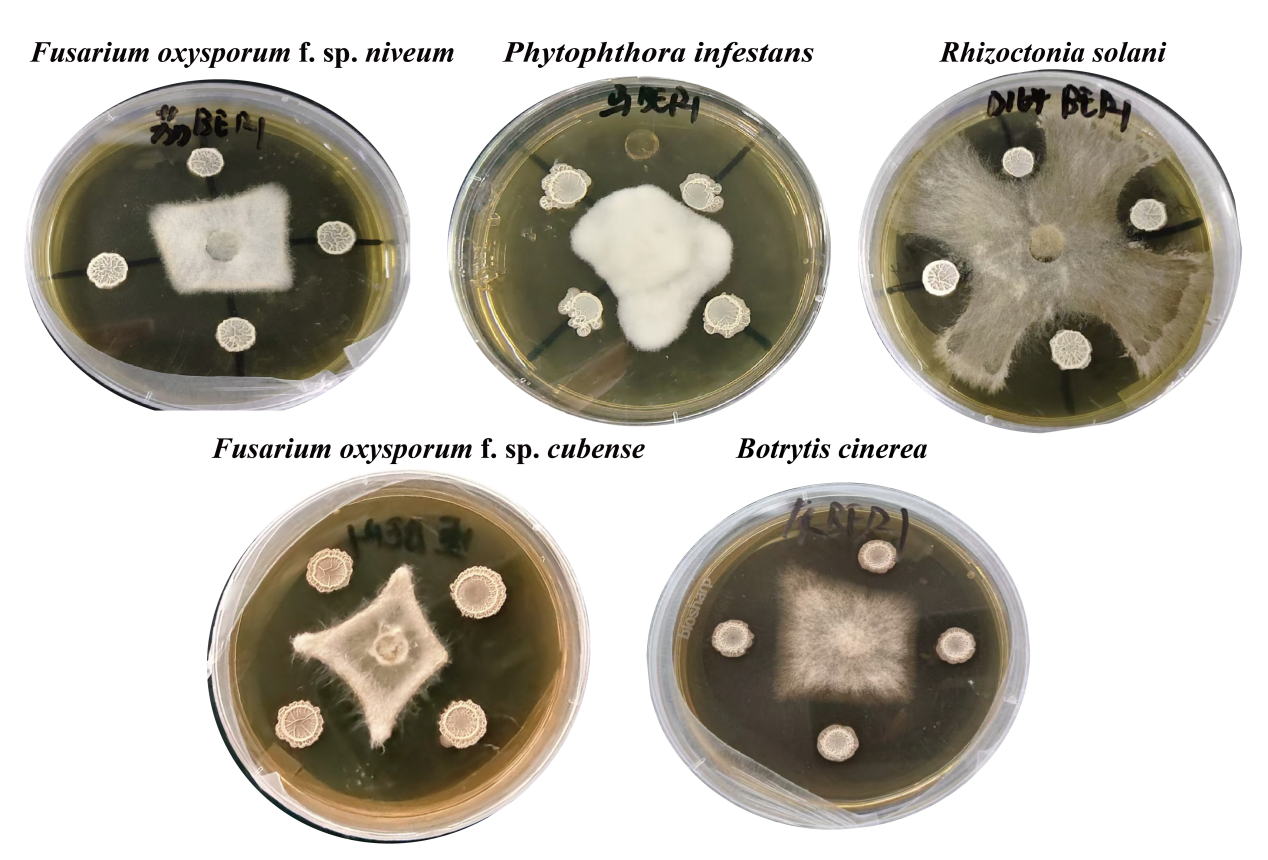


# Figure S1. Antagonistic activity of *Bacillus velezensis* BER1 against five plant pathogenic fungi (*Fusarium oxysporum* f. sp. *Niveum, Phytophthora* *infestans, Rhizoctonia* *solani, Fusarium oxysporum* f. sp. *cubense* and *Botrytis cinerea*) in dual culture assays after 4 days of incubation at 28°C.
